# Enzymatic Synthesis of Anabolic Steroid Glycosides by Glucosyltransferase from *Terribacillus *sp. PAMC 23288

**DOI:** 10.4014/jmb.1911.11057

**Published:** 2019-12-30

**Authors:** Eun-Ji Yu, Tokutaro Yamaguchi, Joo-Ho Lee, A-Rang Lim, Jun Hyuck Lee, Hyun Park, Tae-Jin Oh

**Affiliations:** 1 Department of Life Science and Biochemical Engineering, SunMoon University, Asan 31460, Republic of Korea; 2 Department of Pharmaceutical Engineering and Biotechnology, SunMoon University, Asan 31460, Republic of Korea; 3 Genome-Based BioIT Convergence Institute, Asan 31460, Republic of Korea; 4 Korea Institute of Oriental Medicine, Daejeon 34054, Republic of Korea; 5 Unit of Research for Practical Application, Korea Polar Research Institute, Incheon 21990, Republic of Korea; 6 Department of Polar Sciences, University of Science and Technology, Incheon 21990, Republic of Korea; 7 Division of Biotechnology, College of Life Science and Biotechnology, Korea University, Seoul 02841, Republic of Korea

**Keywords:** Testosterone, nandrolone, glucosylation, neuroprotective activity, rotenone-induced apoptosis, steroid

## Abstract

The application of steroids has steadily increased thanks to their therapeutic effects. However, alternatives are required due their severe side effects; thus, studies on the activities of steroid derivatives are underway. Sugar derivatives of nandrolone, which is used to treat breast cancer, as well as cortisone and prednisone, which reduce inflammation, pain, and edema, are unknown. We linked *O*-glucose to nandrolone and testosterone using UDP-glucosyltransferase (UGT-1) and, then, tested their bioactivities in vitro. Analysis by NMR showed that the derivatives were 17β-nandrolone β-D-glucose and 17β-testosterone β-D-glucose, respectively. The viability was higher and cytotoxicity was evident in PC12 cells incubated with rotenone and, testosterone derivatives, compared to the controls. SH-SY5Y cells incubated with H_2_O_2_ and nandrolone derivatives remained viable and cytotoxicity was attenuated. Both derivatives enhanced neuronal protective effects and increased the amounts of cellular ATP.

## Introduction

Androgen deficiency in older men is mostly the result of an age-associated decrease in testosterone [1, 2]. This is manifested as tardive testicular dysfunction (late-onset hypogonadism, LOH), accompanied by a decrease in memory and concentration as well as increased feelings of despair, dejection, irritation, unease, nervousness, listlessness, and fatigue. Physical symptoms include muscle loss and weakness, decreased bone density, osteoporosis, increased risk of bone fractures, increased visceral fat, sleep disorders, hot flashes, alopecia, skin changes, and a feeling of physical fullness. Sexual dysfunction includes hypoactive sexual desire, erectile dysfunction, and loss of ejaculation [3]. The symptoms of LOH are diverse because testosterone acts on many organs and systems, including the brain, bones, muscles, kidneys, cardiovascular system, testes, penis, prostate, hair follicles, sebaceous glands, hematopoietic cells, and immune system [4-10]. Bone density is maintained by female sex hormones that are synthesized via male hormones; in the brain, testosterone functions as a proneurosteroid [11-25]. Decreased levels of testosterone in the bloodstream might trigger metabolic syndrome and cardiovascular diseases.

Therefore, androgenic replacement therapy (ART) can be applied when testosterone levels fall below a threshold value. Exogenous testosterone or its derivatives can be delivered via patches, gels, intramuscular injections, and ointments, endogenous testosterone can be produced by stimulating testicular Leydig cells [5-7, 26]. However, excessive intake or higher dosages elicit side effects, and a testosterone threshold has been established. Moreover, whether testosterone is the cause or a marker of systemic diseases remains unknown [4]. Further investigations are required to confirm whether blood testosterone levels can be improved or prevented from decreasing with age in men and determine appropriate testosterone forms and dosages.

Testosterone or testosterone esters can be taken as supplements; methyltestosterone and testosterone esters have relatively high activity in the associated organs. Testosterone is inexpensive and testosterone esters are quite simple to synthesize; thus, their application has become widespread. In addition, nandrolone, an anabolic steroid that breaks down proteins, increases bone density, increases red blood cells, muscle growth, and appetite can be ingested as an ester with decanoic acid [27].

These steroids or steroid derivatives are lipophilic and are useful as supplements; however, aqueous forms might be more advantageous for treating systemic diseases. Lipophilic steroids, such as steroid ester derivatives, have been studied in detail; in contrast, studies on hydrophilic steroids are scant. Hydrophilic steroids can be prepared by glycosylation [28], but the yield of conventionally prepared glycosylated products is insufficient and the process is complicated [29, 30]. In addition, investigations into steroid glycosylation might have been avoided because testosterone glycosides have considerably lower local bioactivity than the ester forms [31-33]. Nevertheless, research on hydrophilic steroids, which offer therapeutic advantages for systemic diseases and investigations into androgen deficiency, is needed. From this viewpoint, testosterone and nandrolone glycosides should be investigated as important pharmaceutical agents with the potential to treat diseases associated with steroid hormones.

The glucosyltransferase YjiC derived from an Arctic Bacillus converted over 98% of testosterone and nandrolone to their respective glucosides but not cortisone and prednisone, which are structurally similar to testosterone. While low temperatures need to be maintained for its activity this enzyme can tolerate a wide range of reaction conditions. We optimized the reaction conditions of this enzyme derived from a polar microbe based on reaction kinetics and examined the activity of its steroid glucoside product in brain cells.

## Materials and Methods

### Materials and Solvents

DNA polymerase, dNTP, restriction enzymes, DNA ligase, and T-vector pMD20 were obtained from Takara Clontech (Japan). Isopropyl-β-thiogalactopyranoside (IPTG) and kanamycin were purchased from Duchefa Farma B.V. (The Neterlands); TALON metal affinity resin was obtained from Qiagen (Germany). Steroid substrates containing testosterone, nandrolone, cortisone, and prednisone were purchased from Tokyo Chemical Industry Co., Ltd. (Japan). Rotenone, H_2_O_2_, and 2’,7’-dichlorofluorescin diacetate were purchased from Sigma Chemical (USA). Fetal bovine serum (FBS) and RPMI 1640 medium were purchased from HyClone (USA); 96-well collagen IV-coated and white plates were purchased from BD Biosciences (USA). CellTiter Aqueous One Solution Cell proliferation assay kits (MTS) were purchased from Promega Co. (USA). Luminescence ATP and JC-1 mitochondrial membrane potential detection kits were obtained from PerkinElmer (USA) and Biotium (USA). All other high-grade reagents were obtained from commercial sources.

### Cloning, Overexpression, and Purification

We amplified the UDP-glucosyltransferase gene (UGT-1) of *Terribacillus* sp. PAMC 23288 isolated from an Arctic soil sample using the primers, UGT-1F (5’-GGATCCATGAAACAGCATCATATCACAA-3’) (BamHI, forward) and UGT-1R (5’-CTCGAGTTATTGCGGAACGACAGA-3’) (XhoI, reverse) under amplification conditions of 95°C for 2 min, followed by 35 cycles of 95°C for 1 min, 60°C for 1 min, 72°C for 1 min, and 72°C for 5 min. The PCR mixture (20 μl) contained 10 μl PCR Mix (Noble Bio, Korea), 1 μl each forward primer, reverse primer, and template DNA, and 7 μl distilled water. The UGT-1 gene ligated into the plasmid pET28a(+) was overexpressed in host *E. coli* C41(DE3) cells shaken at 180 rpm in Luria-Bertani medium supplemented with 0.5 mM IPTG at 37°C until cultures reached an optical density of 0.6–0.8 at 600 nm. The cultures were then shaken at 180 rpm for 2 days at 20°C; then, UGT-1 was purified using Co^2+^-affinity resin.

### Enzymatic Assays with Steroids In Vitro

Glucosyl transfer reactions using UGT-1 and various steroids proceeded at 35°C for 3 h in a mixture of 10 μg/ml glucosyltransferase, 100 mM Tris-HCl (pH 8.5) buffer, 10 mM MgCl_2_•6H_2_O, 2 mM UDP-glucose, and 0.4 mM substrate. Reactions were terminated by adding 400 μl methanol to the mixtures and separating them by centrifugation at 12,000 ×*g* for 10 min. The supernatant was analyzed using HPLC and LC-MS. Reaction products were generated in 10–ml volumes containing purified glucosyltransferase (30 μg/ml),100 mM Tris-HCl (pH 8.5) buffer, 10 mM UDP-glucose, 10 mM MgCl_2_•6H_2_O, and 10 mM substrate for 3 h at 35°C. Reactions were terminated by adding two volumes of methanol, followed by centrifugation at 12,000 ×*g* for 10 min. The supernatant was then concentrated by evaporation and purified using preparative HPLC.

### 2.4. Isolation of Steroid Glucosides

Products were analyzed using a Dionex Ultimate 3000 UHPLC+ system (Thermo Fisher Scientific, USA) with a Mightysil 4.6 × 250 mm (5 μm) reverse-phase C18 GP column. The HPLC system comprised an LPG-3400SD pump, an ACC-3000 auto-sampler column compartment, and a DAD-3000 diode array detector. The mobile phase consisted of solution A (HPLC-grade water) and solution B (HPLC-grade acetonitrile). The flow rate was 1.0 ml/min, and the oven temperature was maintained at 30°C. The products were analyzed by increasing the ratio of solution B to solution A from 5 to 8% (0–4 min), 20% (7 min), 40% (10 min), 70% (13 min), and holding at 100% for 18–25 min, and, then, decreasing the ratios to 80% (28 min), 50% (30–33 min), and 5% (38 min). Substrates and their products were confirmed by UV detection at 245 nm.

Compounds were purified by preparative HPLC using a YMC-Pack ODS-AQ C18 column (250 × 20 mm; internal diameter, 10 μm) connected to a UV detector (245 nm); a 35–min binary program comprising ACN 5 to 40% (0–10 min), increase to 100% (10–18 min), hold at 100% (18–25 min), and decrease to 5% (25–35 min) at a flow rate of 10 ml/min was used.

### MS and NMR Analysis

The masses of the glucosylation starting substrates and their products were confirmed by ultra-high performance liquid chromatography electro-spray ionization quadrupole time–of–flight high resolution mass spectrometry (UPLC^®^-ESI-Q-TOF-HRMS) using an ACQUITY UPLC^®^ system (Waters Corporation, USA) coupled to a SYNAPT G2-S mass spectrometer (Waters Corporation). Reaction mixtures were analyzed by UPLC^®^ using a C18 reverse phase column (ACQUITY UPLC^®^ BEH, C18, 1.7 μm) connected to a PDA detector with UV absorbance of 245 nm at 35ºC. Binary mobile phases comprised solvent A (HPLC grade 0.1% tetrafluoroacetic acid (TFA) in water) and solvent B (HPLC grade 0.1% TFA in acetonitrile). The flow rate was maintained at 0.3 ml/min for the entire 12–min gradient program. The proportion of B was increased from 0 to 100% for the initial 7 min, maintained at 100% for 2.5 min, and, then, decreased to 0% for 0.1 min, followed by a constant flow of 0% B for 2.4 min. The ESI-Q-TOF-HRMS polarity, capillary voltage, cone voltage, source temperature, desolvation temperature, desolvation gas flow, and ramp trap collision energy voltage conditions were fixed as follows: ES positive mode, 3 kV, 40 V, 120°C, 350°C, 650°C, and 20–40 V, respectively.

Samples of purified product were dissolved in hexadeuterio-dimethyl-sulfoxide (DMSO-*d6*) for NMR analysis using a Bruker Avance III HD 700 MHz cryogenic spectrometer (Bruker GmbH, Germany). The structures of the glycosylated steroids were determined using one-dimensional (^1^H NMR and ^13^C NMR) and two-dimensional NMR (COrrelation SpectroscopY; COSY), Rotating-frame nuclear Overhauser Effect SpectroscopY (ROESY), Heteronuclear Single Quantum Coherence-Distortionless Enhancement by Polarization Transfer (HSQC-DEPT), and heteronuclear multiple bond connectivity (HMBC) tests. We also used MestReNOVA version 14.0.1 software for processing the MS and NMR data.

### Product Characteristics

**Testosterone glucoside (Product 1).** Table 1 shows the NMR data. ESI-TOP HRMS calculated and found for C_25_H_39_O_7_ [M+H]^+^: 451.26903 and 451.2691, respectively.

**Nandrolone glucoside (Product 2).** Table 2 shows the NMR data. ESI-TOP HRMS calculated and found for C_24_H_37_O_7_ [M+H]^+^: 437.25338 and 437.2537, respectively.

### Cell Cytotoxicity and Protection Assays of Steroid Glucosides

**Testosterone glucoside (Product 1).** Rat PC12 cells (derived from a pheochromocytoma of the rat adrenal medulla) purchased from the Korean Cell Line Bank (Korea) were cultured in RPMI 1640 (Gibco, USA) medium containing 10% fetal bovine serum at 37°C in a 5% CO_2_ atmosphere. The cells were then seeded at a density of 1 × 10^4^/well in RPMI 1640 containing 10% FBS in 96-well collagen IV-coated plates and incubated at 37°C for 24 h. Various concentrations of testosterone glucoside were added, and the plates were incubated at 37°C for a further 24 h. Cell viability was determined using MTS assays (Promega Co.) according to the manufacturer’s instructions. The medium was discarded; then, RPMI (200 μl) containing MTS was added to each well, and the plates were incubated at 37°C for 1 h. The amount of formazan in the wells was assayed at 490 nm using a microplate fluorometer (Molecular Devices, USA). Protective effects were assayed by incubating PC12 cells with testosterone glucoside in 96-well collagen IV-coated plates for 24 h and, then, with 10 μM rotenone for 1 h.

**Nandrolone glucoside (Product 2).** SH-SY5Y neuroblastoma cells (American Type Culture Corporation (ATCC), USA) in Dulbecco’s modified Eagle’s medium (Gibco) supplemented with 10% FBS (Gibco) and 100 U/ml penicillin were seeded at a density of 1 × 10^4^/well and incubated at 37°C for 24 h. Various concentrations of nandrolone glucoside were then added, and the cells were incubated again at 37°C for 24 h. Cell viability was determined using MTS assays as described above. The medium was removed and 200 μl DMEM containing MTS was added to each well; then, the cells were incubated at 37°C for 1 h. Formazan production was assayed at 490 nm using a microplate fluorometer (Molecular Devices). Protective effects were assessed by incubating SH-SY5Y cells with nandrolone glucoside for 24 h, adding 100 μM H_2_O_2_, and incubating them for another 1 h. Cell viability was determined as MTS reduction to formazan as described by the manufacturer.

### Measurement of Cellular ATP

Measuring light intensity using a luminometer permits direct quantitation of ATP; here, ATPlite (Perkin Elmer Inc., USA) was used. Seeded cells were incubated with steroid glucosides for 24 h; then, 10 μM rotenone was added and the cells were incubated for 1 h. Cells were then lysed using cell lysis buffer and incubated with substrates. Total cellular ATP content, determined using luminescence ATP detection kits and a luminometer (Berthold Technologies, USA), is expressed as the ratio (%) of untreated cells (control).

### Statistical Analysis

All measurements were made in triplicate and all data are presented as mean ± standard error of the mean (SEM). The results were subjected to analysis of variance (ANOVA) using Tukey’s multiple comparison test to analyze differences, and *p* < 0.05 was considered to be significant.

## Results and Discussion

### Sequence Analysis, Cloning, and Overexpression of UGT-1

**Fig. 1:**
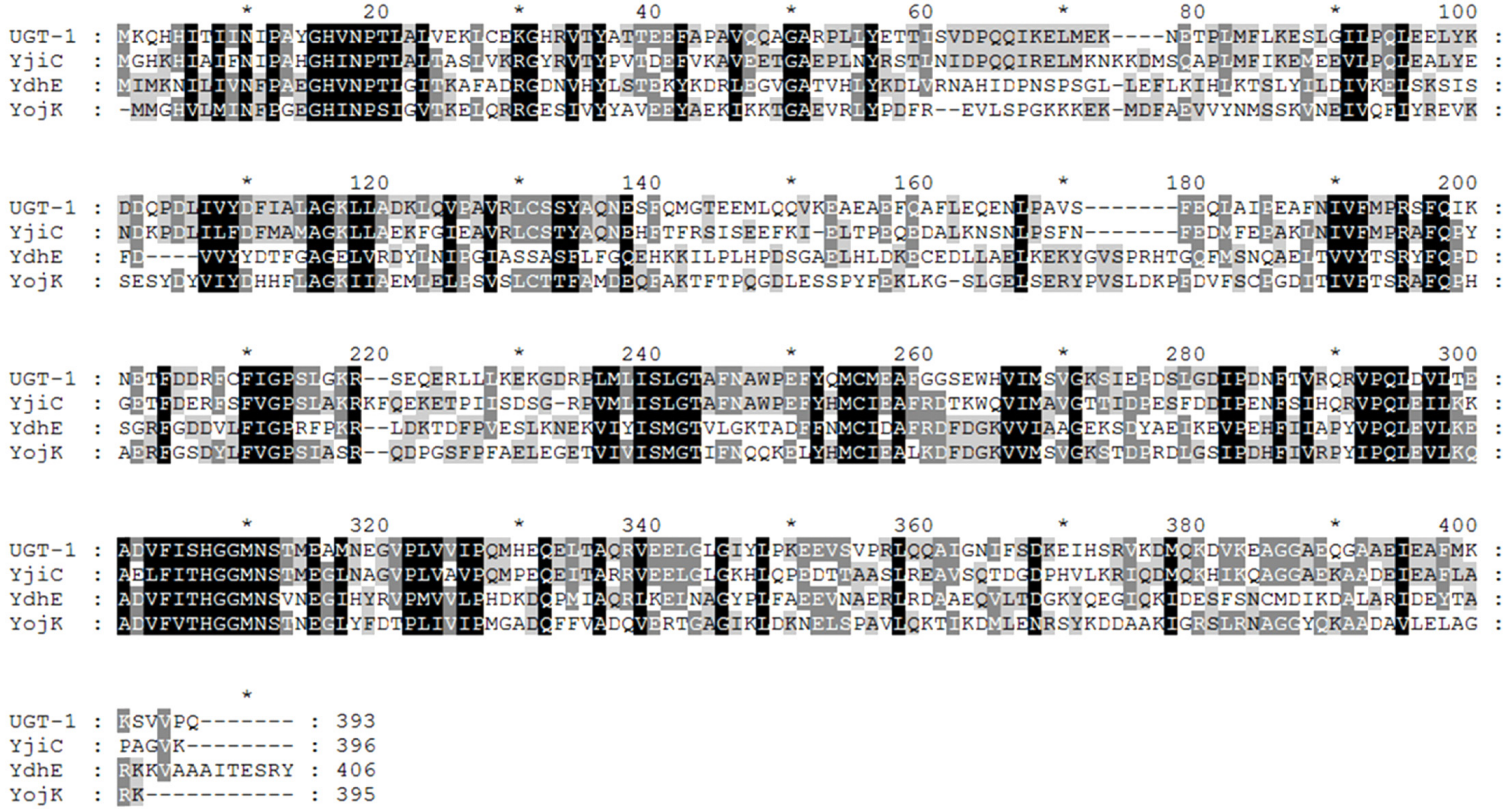
Multiple amino acid sequence alignment of putative glucosyltransferase ClustalX and GeneDoc. Black columns, conserved residues between UGT-1 and other UGT.

**Fig. 2:**
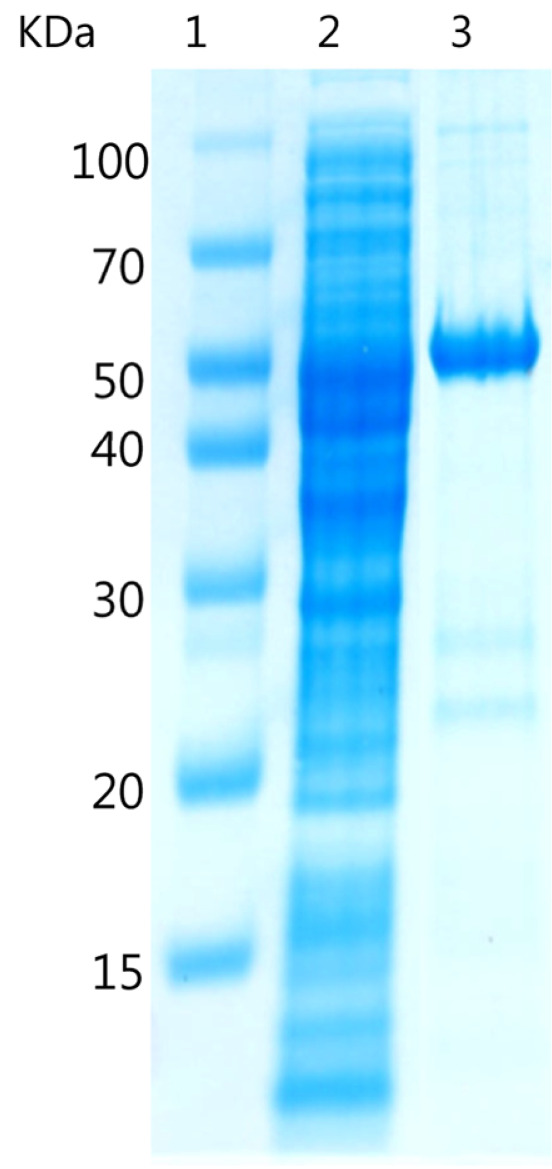
Analysis of purified UGT-1 by SDS-PAGE. Lane 1, molecular mass standards; lane 2, total cell extract of induced *E. coli* C41(DE3) cells; lane 3, UGT-1 purified by affinity chromatography with a Co^2+^ resin.

We aligned multiple sequences using the ClustalX 2.1 program and shaded the aligned sequences using the GeneDoc 2.7 program. YjiC, YdhE, and YojK used for sequence alignment are glucosyltransferases derived from *Bacillus licheniformis* (DSM 13, ATCC 14580), which is available in the NCBI database (GenBank Accession No. CP000002) [34]. Sequencing revealed that our UGT-1 from *Terribacillus* sp. had 52.4%, 31.3%, and 28.2% identity with YjiC, YojK, and YdhE glucosyltransferases, respectively. It also contained the UGP-binding domain found in typical GT (Fig. 1). Heterologous overexpression of UGT-1 in *E. coli* C41(DE3) resulted in a significant yield of target protein with a confirmed molecular weight of 44.2 kDa determined by SDS-PAGE. This was consistent with the predicted molecular mass (Fig. 2). The UGT-1 investigated herein should react not only with known steroids [28] but also with flavonoid substrates that react with YjiC because of its high identity with YjiC [35].

### Steroid Biotransformation

Unlike other anabolic-androgenic steroids (AAS), nandrolone does not accumulate in male androgenic tissues, such as the scalp, skin, and prostate; thus, its deleterious effects are reduced in these tissues [36]. Nandrolone and testosterone are androgenic hormones with similar structures, and both are anabolic steroids [37, 38]. Testosterone plays important roles in promoting the development of reproductive tissues and secondary sexual traits, such as increased hair growth, as well as muscle and bone mass in male humans [38]. It is involved in health, well-being, and the prevention of osteoporosis [39, 40]. In addition to its natural roles, exogenous testosterone is used to counteract decreased endogenous levels in men and elderly persons, treat transsexulas, and treat women with breast cancer [41].

Cortisone is a corticosteroid hormone associated with cortisol during pregnancy; it is the main hormone released by the adrenal glands in response to stress. It is used to treat various diseases by inhibiting the immune system and reducing inflammation, pain, and edema at sites of injury [42, 43]. Prednisone is a glucocorticoid that is mostly used to suppress the immune system and reduce inflammation associated with pathological states such as asthma, COPD, and rheumatic disease. It is also combined with other steroids to treat high blood pressure caused by cancer and adrenal insufficiency [44]. However, long-term administration is associated with side effects; moreover, it is converted to biologically active prednisolone in the liver, which causes changes in gene expression [45, 46].

**Fig. 3:**
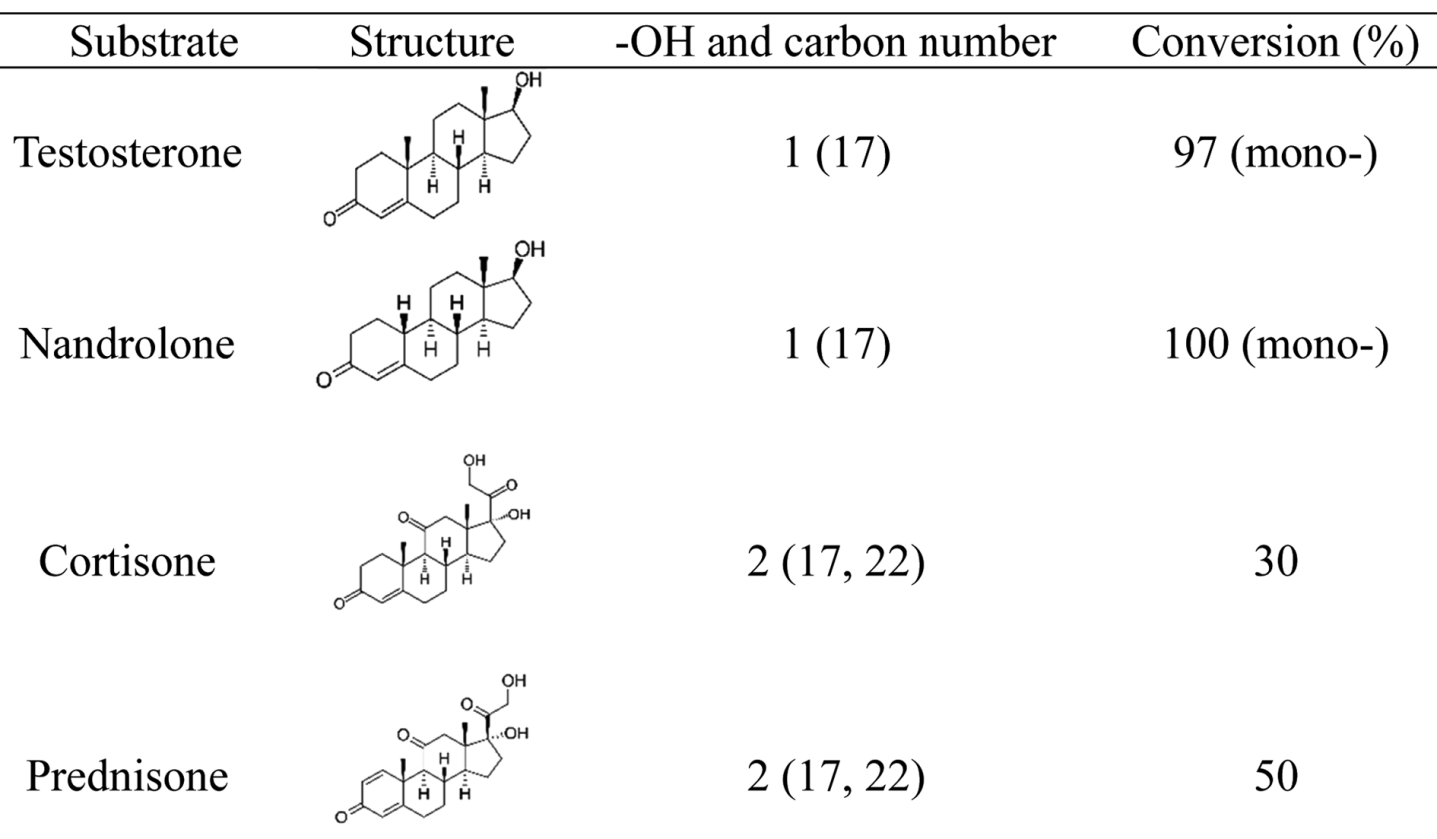
Carbon number, -OH, and conversion rates of steroid substrates.

Corticosterone is glucosylated by UGT-1, which attaches a sugar to C21 [28]. Sugars are attached to testosterone, nandrolone, cortisone, and prednisone at terminal hydroxyl groups. Accordingly, UTP binds glucose to substrates in bacterial GT systems. Fig. 3 shows that the UGT-1 enzyme bound glucose to the hydroxyl groups of the steroid substrates. Testosterone, nandrolone, cortisone, and prednisone were glucosylated at 14.3, 14.1, 13.7, and 13.6 min after the reaction with UGT-1, according to HPLC/LC-MS analysis, confirming its activity (Fig. S1). The masses of testosterone and nandrolone peaks analyzed by UPLC-ESI-Q-TOF-HRMS were 451.2691 and 437.2537, respectively, indicating mono-glucosylated products.

### Determination of the Testosterone and Nandrolone Glycoside Structures (Products 1 and 2)

**Fig. 4:**
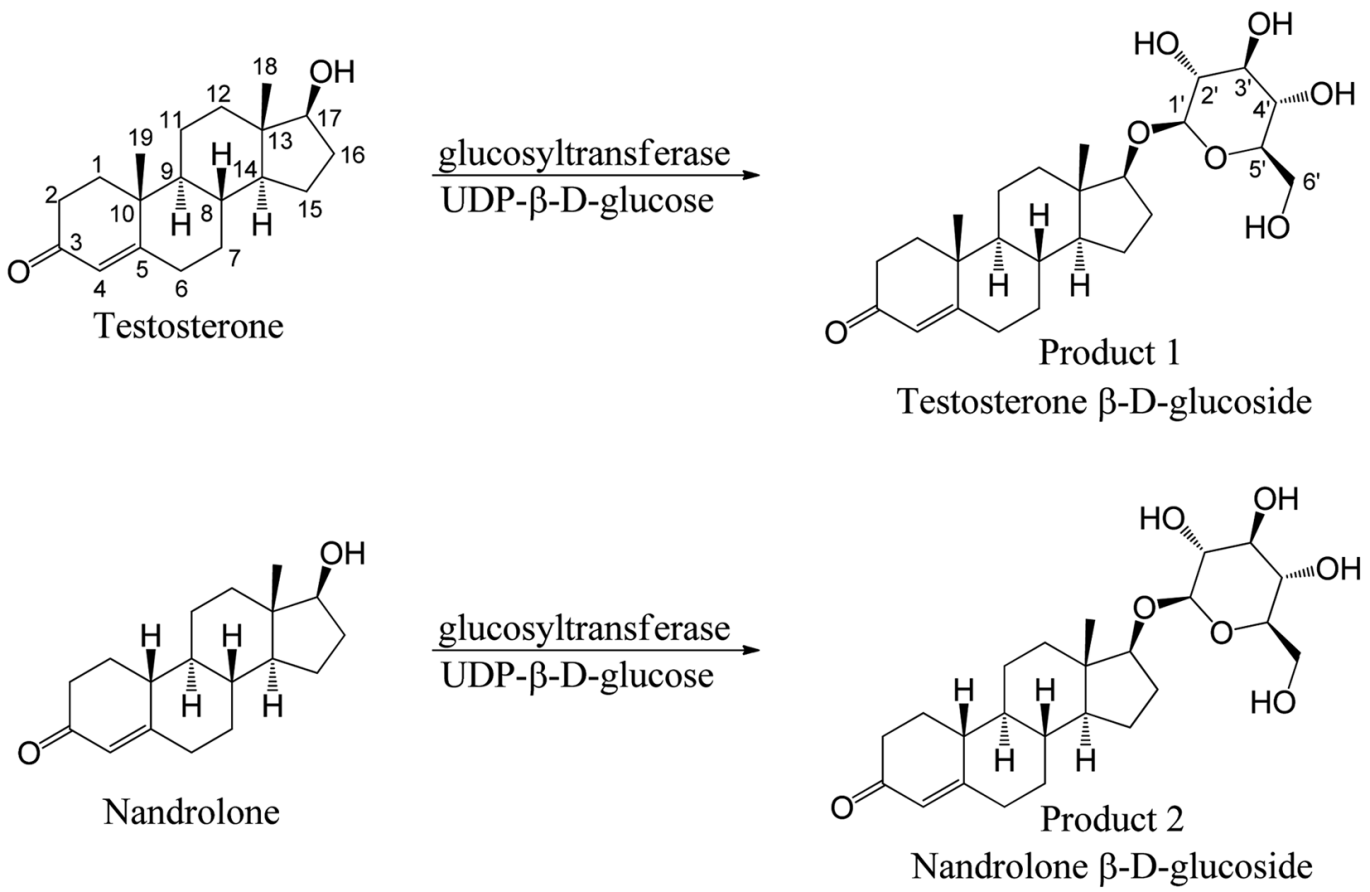
Scheme of glucosyltransferase conversion of testosterone and nandrolone.

**Fig. 5:**
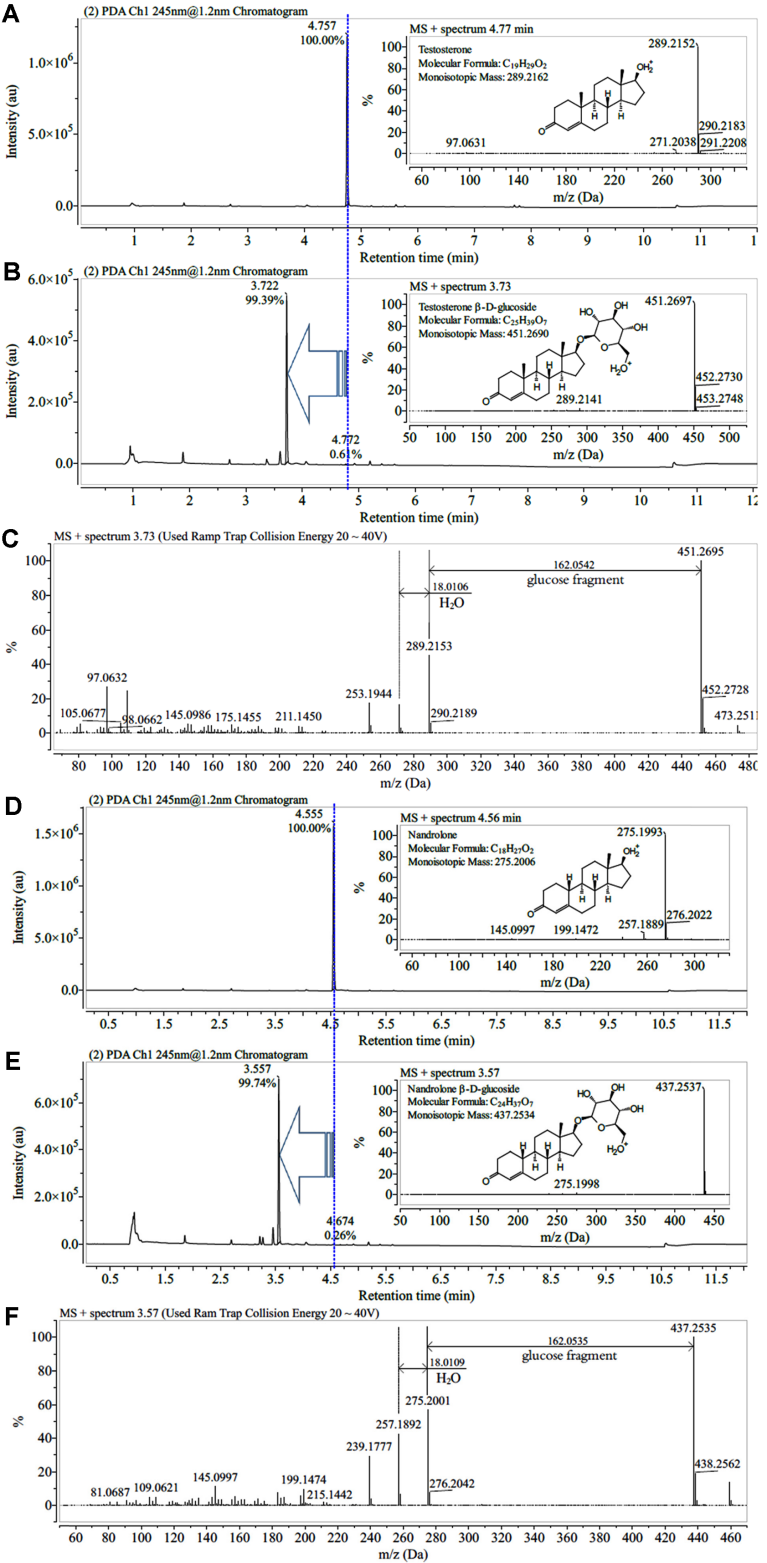
Chromatography and mass spectrum findings. (**A** and **D**) Chromatograms before biotransformation. (**B** and **E**) Chromatograms of products 1 and 2 after testosterone and nandrolone biotransformation. Conversion rates are both > 98%. (**C** and **F**) Mass spectra of products 1 and 2 at ramp trap collision energy of 20–40 V. Fragments of glucose and water are lost from products 1 and 2.

The UPLC-ESI-Q-TOF-HRMS analysis of the testosterone and nandrolone biotransformation products (Fig. 4) revealed glucoside–like peaks at 3.73 and 3.58 min with m/z = 451.2697 (C_25_H_39_O_7_^+^: calculated mass value m/z = 451.2690) and m/z = 437.2537 (C_24_H_37_O_7_^+^: calculated mass value m/z = 437.253), respectively, which were the same [M+1]^+^ as the actual masses of testosterone and nandrolone glucoside. Furthermore, MS spectra using ramp trap collision energy at 20–40 volts showed product ions formed by the loss of the glucoside moiety without or with the loss of one or two water molecules (Fig. 5C and 5F). These MS spectra supported the notion that a glucose moiety is linked to each hydroxyl group of testosterone and nandrolone by glycosylation with glucosyltransferase; these findings corresponded to the NMR findings (ROESY and HMBC spectra, Figs. S20, S22, S27, and S29).

**Fig. 6:**
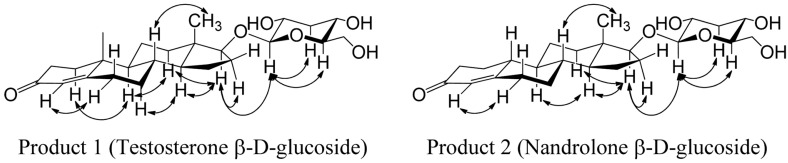
ROESY spectra of products 1 and 2. Spectra show correlation peaks for each hydrogen.

The structures of the glycosylated products 1 and 2 were elucidated using one-dimensional (^1^H NMR and ^13^C NMR) and two-dimensional NMR (COSY, ROESY, HSQC-DEPT, and HMBC). As NMR data for DMSO-*d*6 as the solvent are not available, we fully elucidated the structures of testosterone and nandrolone using 1D and 2D NMR spectroscopy (Figs. S2–S29, Tables 1 and 2). The ^1^H NMR, ^13^C NMR, and the HSQC-DEPT study of the purified reaction products revealed an anomeric proton and a carbon at chemical shifts of δ = 4.16 ppm (d, *J* = 7.8 Hz, ^1^H, H-1') and δ = 103.08 ppm, respectively, for product 1 and δ = 4.17 ppm (d, *J* = 7.8 Hz, ^1^H, H-1') and δ = 103.00 ppm, respectively, for product 2. Furthermore, the coupling constants were both 7.8 Hz; the ROESY analysis showed correlations between the anomeric protons and the H-3’ and H-5’ protons because axial conformation (data in Fig. 6 were supported by correlation peaks in Figs. S20 and S27) represented the anomeric protons of products 1 and 2 with a beta (β) configuration of the glucose moiety, respectively, whereas other spectra were evident in the glucose region between δ = (2.9–3.7) ppm (Figs. S16 and S23). Other protons of the testosterone and nandrolone standards and their products precisely matched when compared with the ^1^H NMR and ^13^C NMR of sample testosterone and nandrolone (Tables 1 and 2 and Figs. S2–S29). We further confirmed the glycosylation position using 2D-NMR. The ROESY spectra showed correlations between the anomeric protons H-1’ of products 1 and 2 and H-17 (δ= 3.64 and 3.67 ppm for products 1 and 2, respectively) providing a clue regarding the attachment of glucoses moieties to the C-17 hydroxyl group of testosterone and nandrolone shown in Fig. 6 (Figs. S20 and S27).

These results were further supported by COSY, HSQC-DEPT, and HMBC findings showing correlations between protons and other protons as well as carbons. The HMBC findings (Figs. S22 and S29) showed distinct correlations between the anomeric protons (H-1' of products 1 and 2) and C-17 (δ = 87.25 and 87.27 for products 1 and 2, respectively) of the steroid skeleton. These NMR findings confirmed the glycosylation position at the C-17 hydroxyl group. Thus, products 1 and 2 were confirmed as testosterone β-D-glucoside and nandrolone β-D-glucoside, respectively. These NMR data have not been hitherto obtained using DMSO-*d*6 as the solvent. Furthermore, nandrolone β-D-glucoside is a novel glucoside derivative.

### Cell Cytotoxicity and Protection Assays

**Fig. 7:**
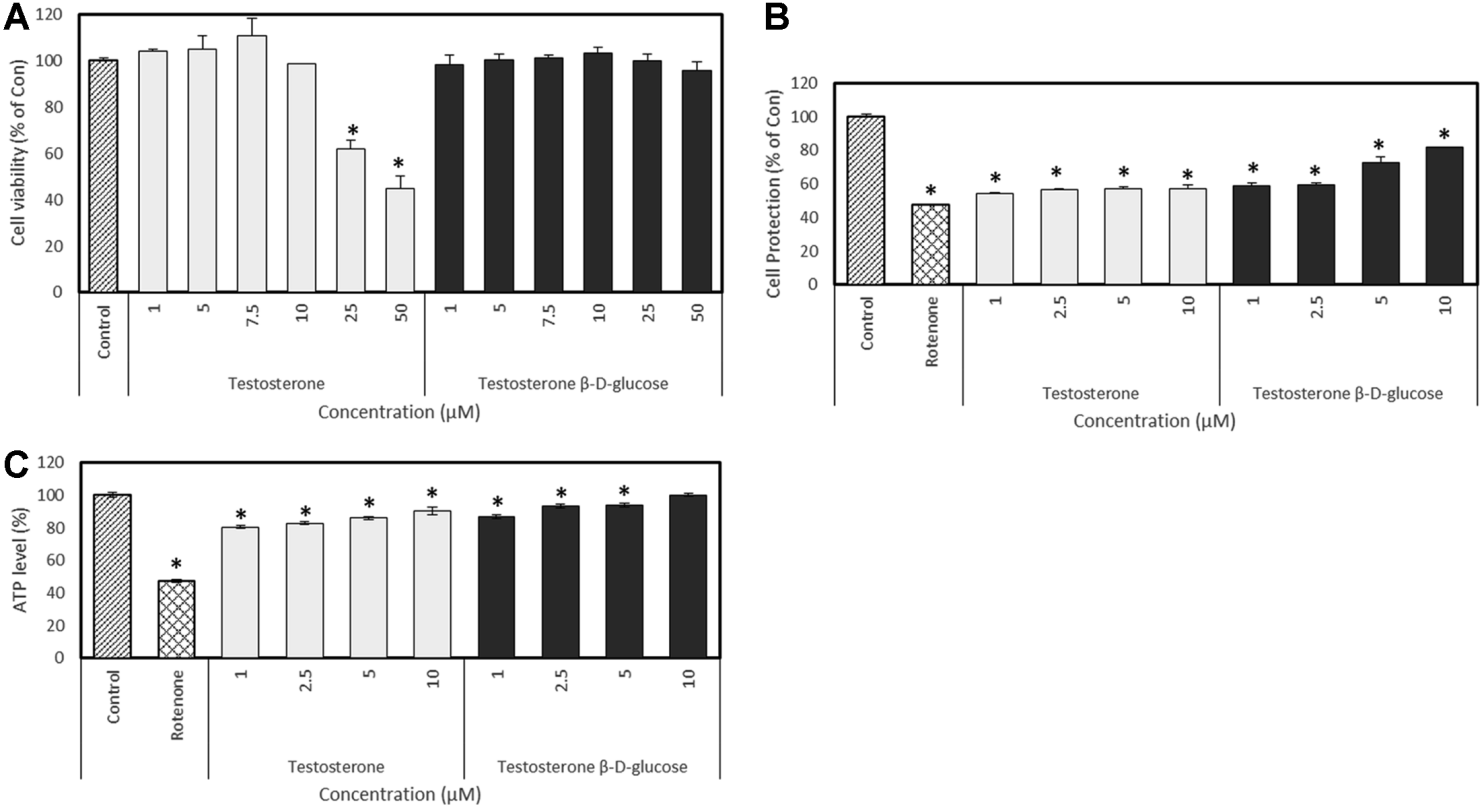
Effects of testostrone and product 1 on PC12 cells. (**A**) Cell viability. (**B**) Protection of cells against rotenone damage. (**C**) Amounts of intracellular ATP in cells incubated with rotenone. Data are reported as percentages of the control. *p < 0.05 compared with the result of control.

Rotenone, a mitochondrial complex I (NADH-quinone reductase) inhibitor, has been used to experimentally induce and model the cellular metabolic disorders and damage associated with Parkinson’s disease (PD), LHON, and the retina [47, 48]. Rotenone toxicity is associated with adenosine triphosphate (ATP), which induces endoplasmic reticulum (ER) stress in some types of cells and causes apoptosis before stressors become apparent [47-50]. Incubation with various concentrations of rotenone for 1h induced significant cytotoxicity in PC12 cells compared to the control, as evidenced by a survival rate of 50% at 10 μM (data not shown). Fig. 7A shows that testosterone decreased PC12 cell viability by 44.6%, compared to the control (100%). However, testosterone derivatives were not highly cytotoxic to PC12 cells (survival, 95.8 ± 4.0% at 50 μM) compared to the controls (Fig. 7A).

**Fig. 8:**
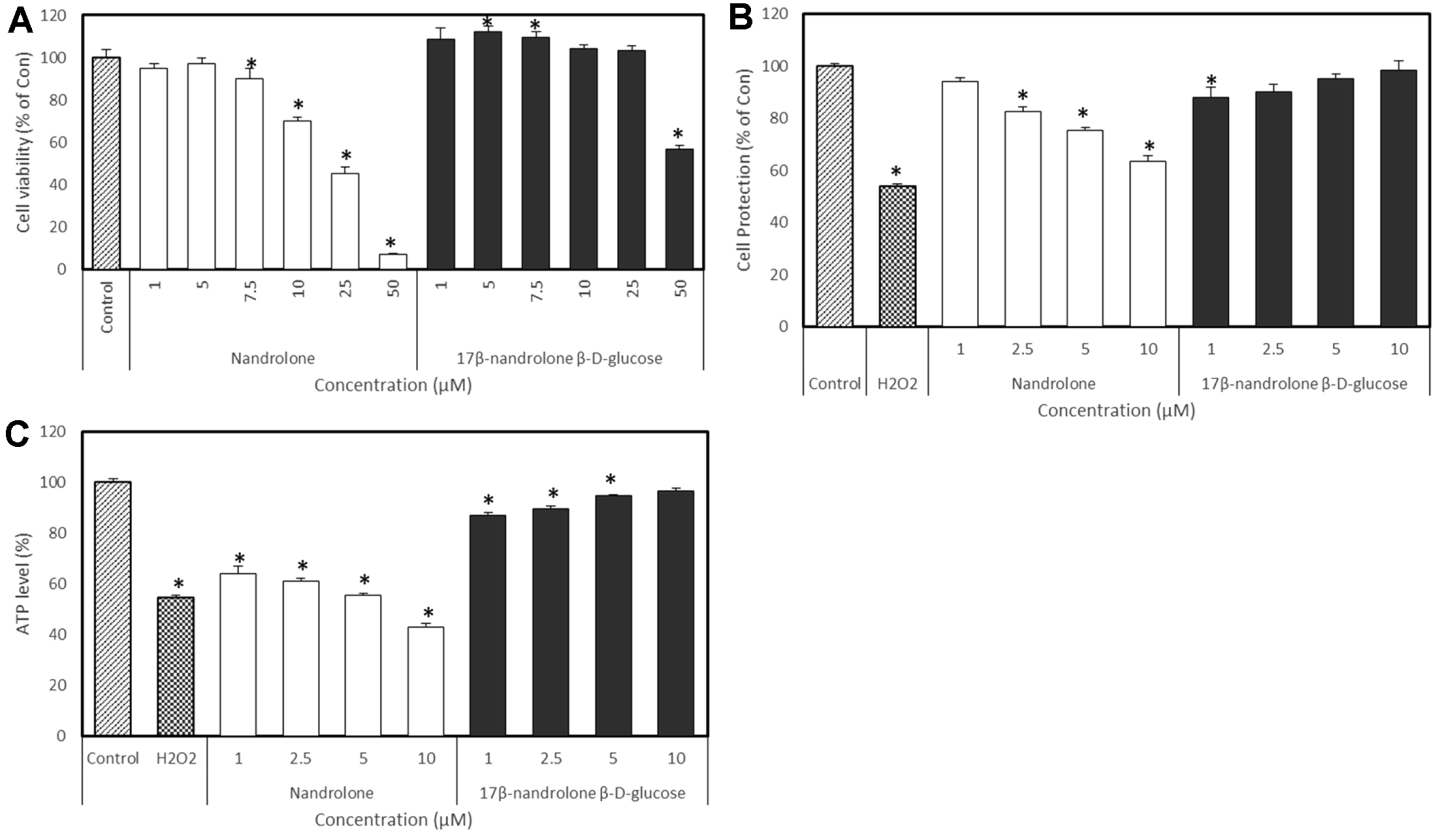
Effects of nandrolone and product 2 on SH-SY5Y neuroblastoma cells. (**A**) Cell viability. (**B**) Protection of cells against H_2_O_2_ damage. (**C**) Amounts of intracellular ATP in cells incubated with H_2_O_2_. Data are reported as percentages of the control. *p < 0.05 compared with the result of control.

We similarly investigated the effects of steroids and their derivatives in SH-SY5Y cells incubated with H_2_O_2_. We found that 100 μM of H_2_O_2_ decreased the survival of SK-N-SH cells by 50% compared to the controls. Nandrolone reduced cell viability, whereas 17β-nandrolone β-D-glucose had no effects on cell viability up to 25 μM. Cells pretreated with 17β-nandrolone β-D-glucose remained viable in the presence of 100 μM H_2_O_2_ (Fig. 8B). Therefore, 17β-nandrolone β-D-glucose attenuated cell toxicity, compared with nandrolone.

### Cellular Energy Metabolism (Total Cellular ATP)

We assessed the amounts of intracellular ATP in PC12 cells that were induced by rotenone and, then, incubated with or without testosterone and its derivatives. Rotenone (10 μM) reduced cellular ATP by 47.5 ± 1.4% in PC12 cells, whereas testosterone derivatives dose-dependently increased cellular ATP compared to testosterone, indicating a protective effect against neuronal cell damage caused by rotenone (Fig. 7C). We also assessed the amounts of intracellular ATP in SH-SY5Y cells induced by H_2_O_2_ and, then, incubated with or without nandrolone and its derivatives. Incubating SH-SY5Y cells with H_2_O_2_ reduced intracellular ATP by 54.6% (Fig. 8C), whereas nandrolone derivatives dose-dependently increased intracellular ATP, compared with nandrolone. The present study found that nandrolone and testosterone glucosides have low cytotoxicity and can protect cells. Therefore, these derivatives might be useful in terms of developing new therapeutics.

## Supplementary material

Supplementary data for this paper are available on-line only at http://jmb.or.kr.



## Figures and Tables

**Table 1:** ^13^C and ^1^H NMR date of testosterone and testosterone β-D-glucoside (product 1).

^13^C NMR				^1^H NMR				

CDCl_3_^a^		DMSO-*d*_6_		CDCl_3_^a^		DMSO-*d*_6_		

#	Chemical shift δ (ppm)			Chemical shift δ (ppm)				*J*_HH_ (Hz)
1	35.67	35.15	35.15	Hα	1.70	1.59	1.58	ddd, 14.1, 13.5, 4.4
				Hβ	2.03	1.96	1.96	ddd, 13.4, 4.9, 3.1
2	33.94	33.62	33.62	Hα	2.35	2.15	2.15	ddd, 16.7, 3.8, 3.8
				Hβ	2.39	2.40	2.39	m
3	199.60	198.02	198.06				-	
4	123.85	123.13	123.16	H	5.73	5.62	5.62	d, 2.1
5	171.35	171.07	171.04				-	
6	32.80	31.98	31.95	Hα	2.28	2.23	2.23	ddd, 14.5, 4.1, 2.3
				Hβ	2.39	2.38	2.38	m
7	31.55	31.30	31.25	Hα	1.01	0.91	0.92	m
				Hβ	1.85	1.76	1.76	dddd, 12.8, 5.7, 2.7, 2.7
8	35.73	35.08	34.86	H	1.58	1.52	1.53	m
9	53.93	53.44	53.29	H	0.93	0.86	0.87	ddd, 12.2, 10.6, 3.9
10	38.67	38.24	38.22				-	
11	20.65	20.25	20.18	Hα	1.60	1.51	1.49	m
				Hβ	1.44	1.36	1.37	dddd, 13.2, 13.2, 13.2, 4.1
12	36.44	36.29	36.75	Hα	1.09	0.97	1.12	dd, 13.0, 4.1
				Hβ	1.86	1.75	1.92	m
13	42.82	42.38	42.55				-	
14	50.49	50.00	49.87	H	0.98	0.89	0.93	m
15	23.34	23.01	22.82	Hα	1.63	1.50	1.51	m
				Hβ	1.31	1.20	1.21	dddd, 12.9, 12.6, 11.9, 5.0
16	30.41	29.81	28.45	Hα	2.08	1.83	1.91	m
				Hβ	1.47	1.35	1.52	m
17	81.56	79.85	86.82	H	3.66	3.43	3.64	m
18	11.06	11.21	11.36	H3	0.80	0.68	0.79	s
19	17.42	16.92	16.93	H3	1.20	1.14	1.14	s

				17OH	2.42	4.46		

1'			103.08	H			4.16	d, 7.8
2'			73.69	H			2.90	dd, 9.0, 7.8
3'			76.89	H			3.10	dd, 8.8, 8.8
4'			70.14	H			3.02	m
5'			76.86	H			3.02	m
6'			61.12	Ha			3.42	dd, 11.7, 5.5
				Hb			3.64	m

^a^Chemical shifts in CDCl_3_. Referred from D.N. Kirk, H.C. Toms, C. Douglas, K.A. White, K.E. Smith, S. Latif, R.W.P. Hub-bard A survey of the high-field 1H NMR spectra of the steroid hormones, their hydroxylated derivatives, and related compounds, J. Chem. Soc. Perkin Trans. 2. 2 (1990) 1567–1594. doi:10.1039/p29900001567 and SDBSWeb : https://sdbs.db.aist.go.jp (National Institute of Advanced Industrial Science and Technology, date of access: 2018.10.16.). All samples using DMSO-*d*6 solvent were measured by ^13^C NMR and ^1^H NMR spectrometry in terms of 176 and 700 MHz frequency, respectively, in DMSO-*d6*. Assignments from ^1^H-^1^H COSY, ROESY, HSQC-DEPT, and HMQC.

**Table 2:** ^13^C and ^1^H NMR date of nandrolone and nandroloneβ-_D_-glucoside(product 2).

^13^C NMR				^1^H NMR				

CDCl_3_^a^		DMSO-*d*_6_		CDCl_3_^a^		DMSO-*d*_6_		

#	Chemical shift δ (ppm)			Chemical shift δ (ppm)				*J*_HH_ (Hz)
1	26.61	26.15	26.16	Hα	1.56	1.43	1.42	m
				Hβ	2.28	2.19	2.19	m
2	36.50	36.18	36.17	Hα	2.40	2.21	2.22	m
				Hβ	2.25	2.21	2.22	m
3	199.90	198.43	198.45				-	
4	124.57	123.73	123.75	H	2.83	5.72	5.72	dd, 1.9, 1.9
5	166.66	166.91	166.84				-	
6	35.50	34.66	34.62	Hα	2.47	2.42	2.42	ddd, 14.5, 4.0, 2.5
				Hβ	2.28	2.26	2.25	dddd, 14.7, 14.0, 5.2, 2.0
7	30.72	30.39	30.30	Hα	1.06	0.94	0.95	dddd, 13.7, 12.9, 11.2, 3.9
				Hβ	1.84	1.74	1.74	m
8	40.54	39.79	39.51	H	1.37	1.32	1.33	dddd, 11.2, 11.2, 11.1, 3.1
9	49.61	49.13	48.97	H	0.85	0.77	0.79	dddd, 11.1, 11.0, 10.7, 4.2
10	42.62	41.76	41.70	H	2.10	2.14	2.15	ddd, 10.5, 10.0, 2.4
11	26.16	25.66	25.59	Hα	1.86	1.76	1.76	m
				Hβ	1.27	1.21	1.24	dddd, 13.3, 13.1, 12.3, 3.9
12	36.45	36.32	36.80	Hα	1.11	0.99	1.15	ddd, 13.0, 12.9, 4.0
				Hβ	1.88	1.73	1.90	m
13	43.04	42.60	42.77				-	
14	49.78	49.26	49.12	H	1.01	0.91	0.97	m
15	23.20	22.87	22.87	Hα	1.32	1.50	1.50	m
				Hβ	1.62	1.20	1.22	m
16	30.43	29.79	28.40	Hα	2.07	1.84	1.91	m
				Hβ	1.47	1.34	1.51	m
17	81.66	79.93	86.78	H	3.66	3.45	3.66	dd, 9.0, 8.3
18	11.07	11.21	11.35	H3	0.81	0.69	0.81	s

				17OH	1.70	4.47		

1'			103.00	H			4.17	d, 7.8
2'			73.69	H			2.90	dd, 7.9, 7.9
3'			76.84	H			3.10	dd, 7.9, 7.9
4'			70.12	H			3.02	m
5'			76.84	H			3.02	m
6'			61.11	Ha			3.42	m
				Hb			3.64	m

^a^SDBSWeb : https://sdbs.db.aist.go.jp (National Institute of Advanced Industrial Science and Technology, date of access: 2018.10.16.). All samples using DMSO-*d*6 solvent were measured by ^13^C NMR and ^1^H NMR spectrometry in terms of 176 and 700 MHz frequency, respectively, in DMSO-*d6*. Assignments from ^1^H-^1^H COSY, ROESY, HSQC-DEPT, and HMQC.
