# Supplementary material for: Enzymatic Synthesis of Anabolic Steroid Glycosides by Glucosyltransferase from Terribacillus sp. PAMC 23288
Source: J Microbiol Biotechnol. 2019 Dec 30;30(4):604–14. doi: 10.4014/jmb.1911.11057 (PMC9728329; doi:10.4014/jmb.1911.11057)
Supplement: Supplementary file 1 [file JMB-30-4-604-supple.pdf]

J. Microbiol. Biotechnol.
https://doi.org/10.4014/jmb.1911.11057

Enzymatic synthesis of anabolic steroid glycosides by glucosyltransferase from *Terribacillus* sp. PAMC 23288

Eun-Ji Yu[1,†], Tokutaro Yamaguchi[2,†], Joo-Ho Lee[3], A-Rang Lim[4], Jun Hyuck Lee[5,6], Hyun Park[7,*], and Tae-Jin Oh[1,2,3,*]

[1]Department of Life Science and Biochemical Engineering, SunMoon University, 70 Sunmoon-ro 221, Tangjeong-myeon, Asan-si, Chungnam 31460, Republic of Korea

[2]Department of Pharmaceutical Engineering and Biotechnology, SunMoon University, 70 Sunmoon-ro 221, Tangjeong-myeon, Asan-si, Chungnam 31460, Republic of Korea

[3]Genome-based BioIT Convergence Institute, 70 Sunmoon-ro 221, Tangjeong-myeon, Asan-si, Chungnam 31460, Republic of Korea

[4]Korea Institute of Oriental Medicine, 1672 Yuseongdae-ro, Yuseong-gu, Daejeon, 34054, Republic of Korea

[5]Unit of Research for Practical Application, Korea Polar Research Institute, Incheon 21990, Republic of Korea

[6]Department of Polar Sciences, University of Science and Technology, Incheon 21990, Republic of Korea

[7]Division of Biotechnology, College of Life Science and Biotechnology, Korea University, Seoul 02841, Republic of Korea

[†]These authors contributed equally to this work.

[*]Co-corresponding author: H. Park & T.J. Oh

H. Park, Division of Biotechnology, College of Life Sciences and Biotechnology, Korea University, Seoul 02841, Korea. Tel: +82 2 3290 3051; E-mail: hpark@korea.ac.kr

T.-J. Oh, Department of Pharmaceutical Engineering and Biotechnology, SunMoon University, Asan 31460, Korea. Tel: +82 41 530 2677; E-mail: tjoh3782@sunmoon.ac.kr

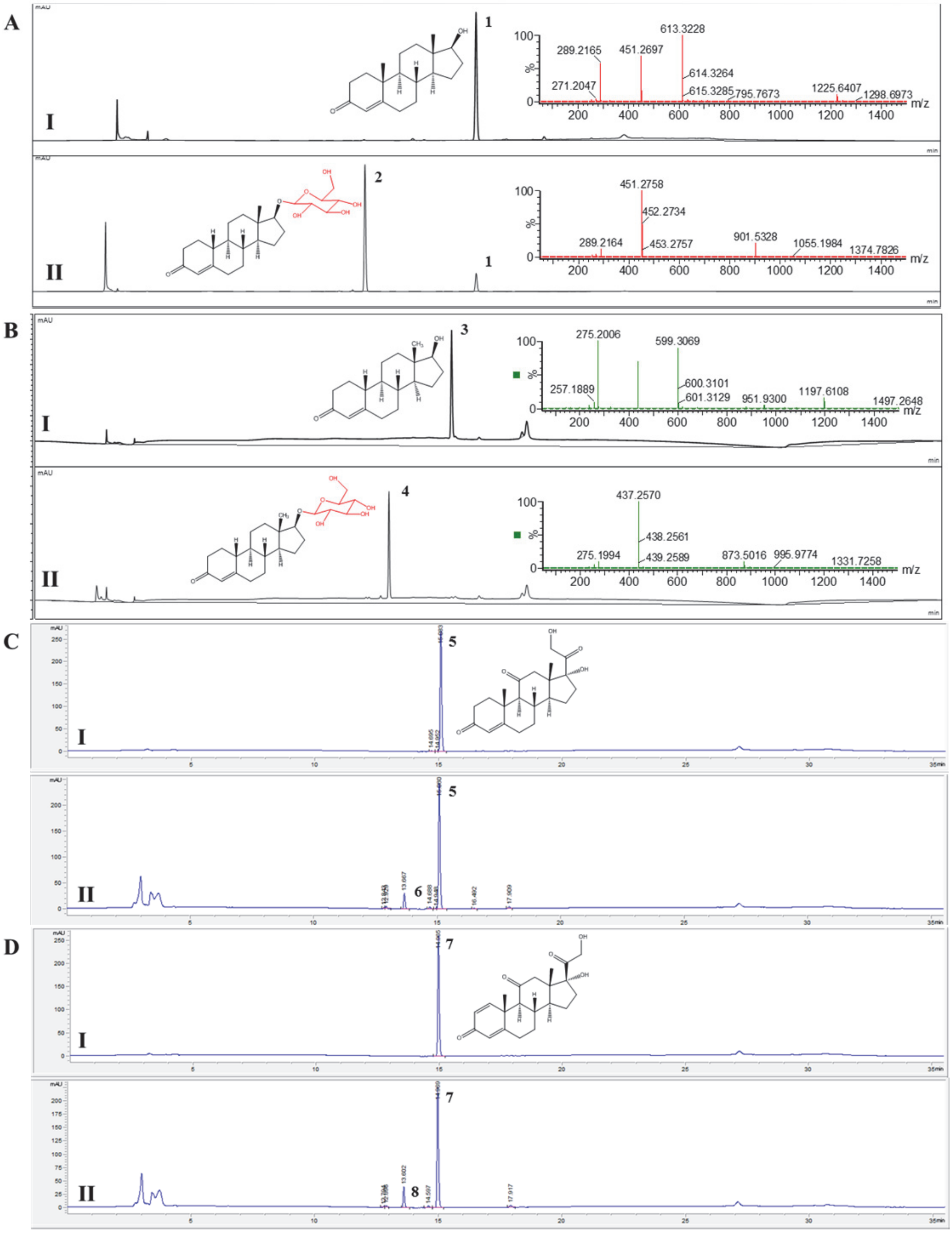

Figure S1. HPLC analysis of the substrates transfer product after biotransformation. Testosterone (A), nandrolone (B), cortisone (C), and prednisone (D) by glucosylation of glucosyltransferase. Insets I and II show the HPLC analysis of the reaction performed in the presence and absence of UGT-1, respectively. 1, testosterone; 2, testosterone β-D-glucose; 3, nandrolone; 4, 17β-nandrolone β-D-glucose; 5, cortisone; 6, product of cortisone; 7, Prednisone; and 8, product of prednisone.

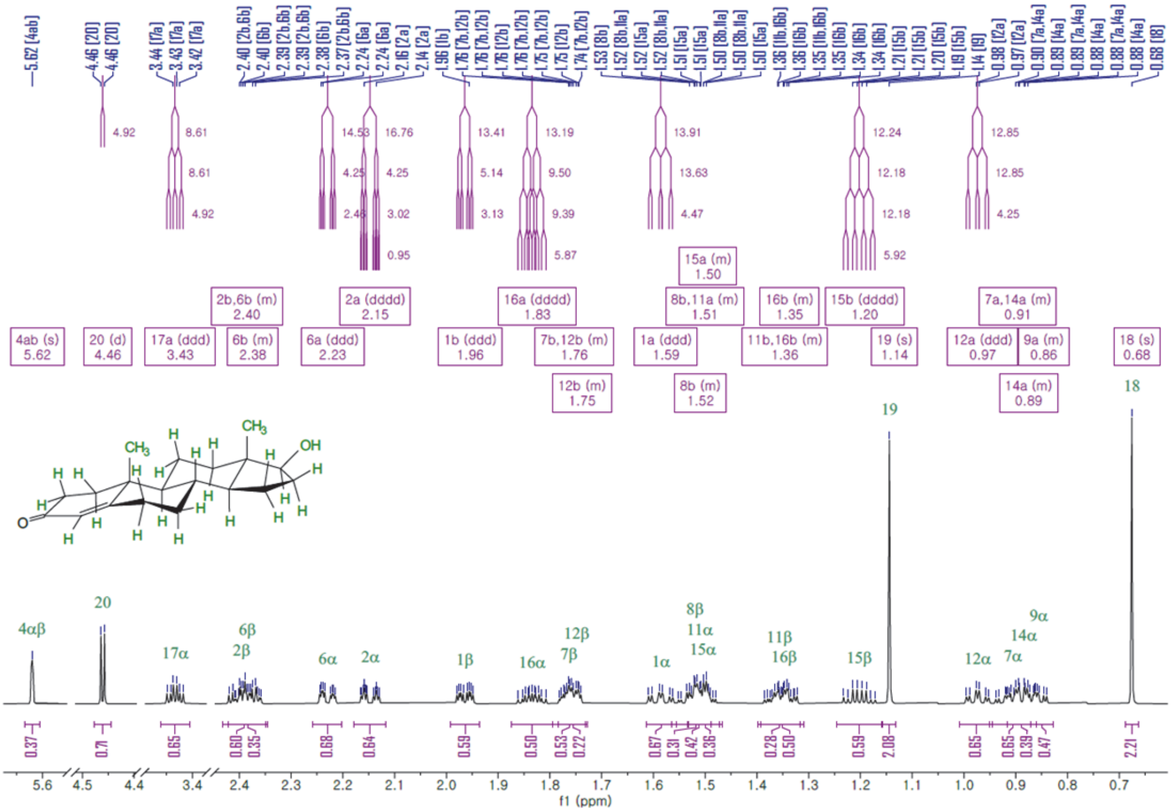

Figure S2. ^{1}H NMR spectra of testosterone standard.

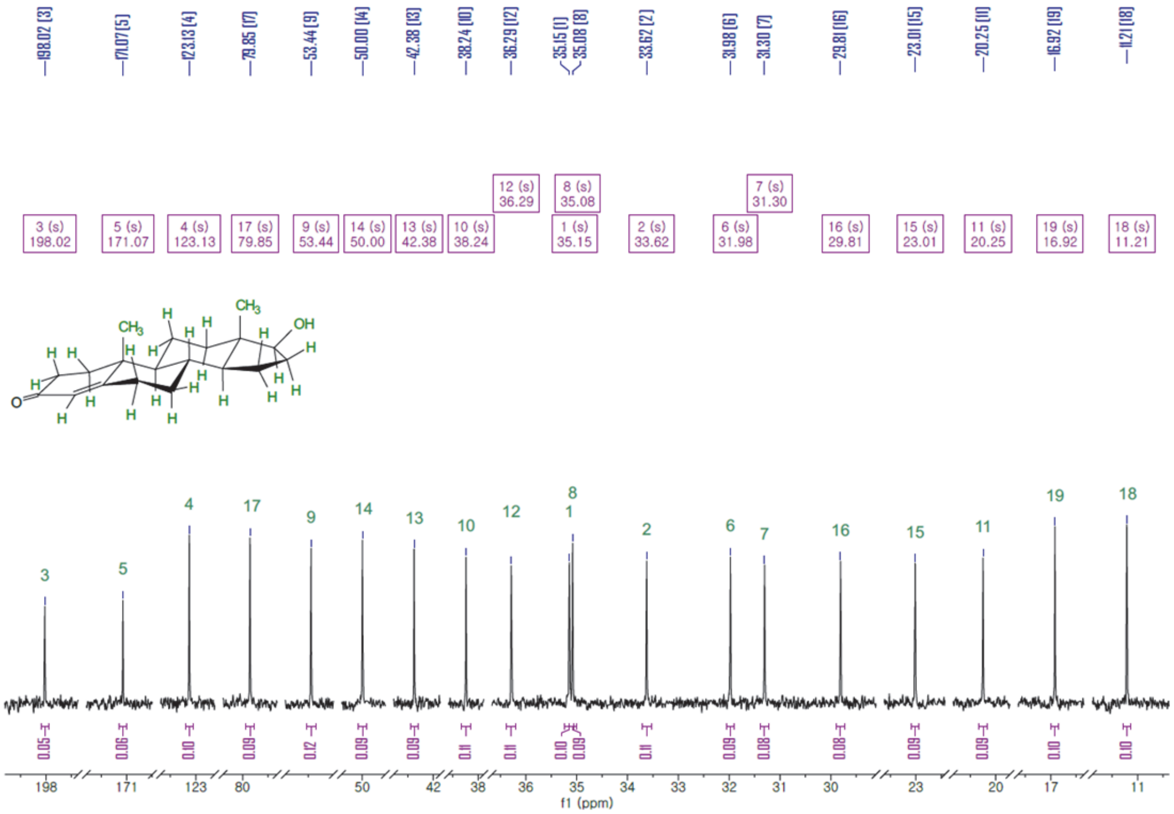

Figure S3. ^{13}C NMR spectra of testosterone standard.

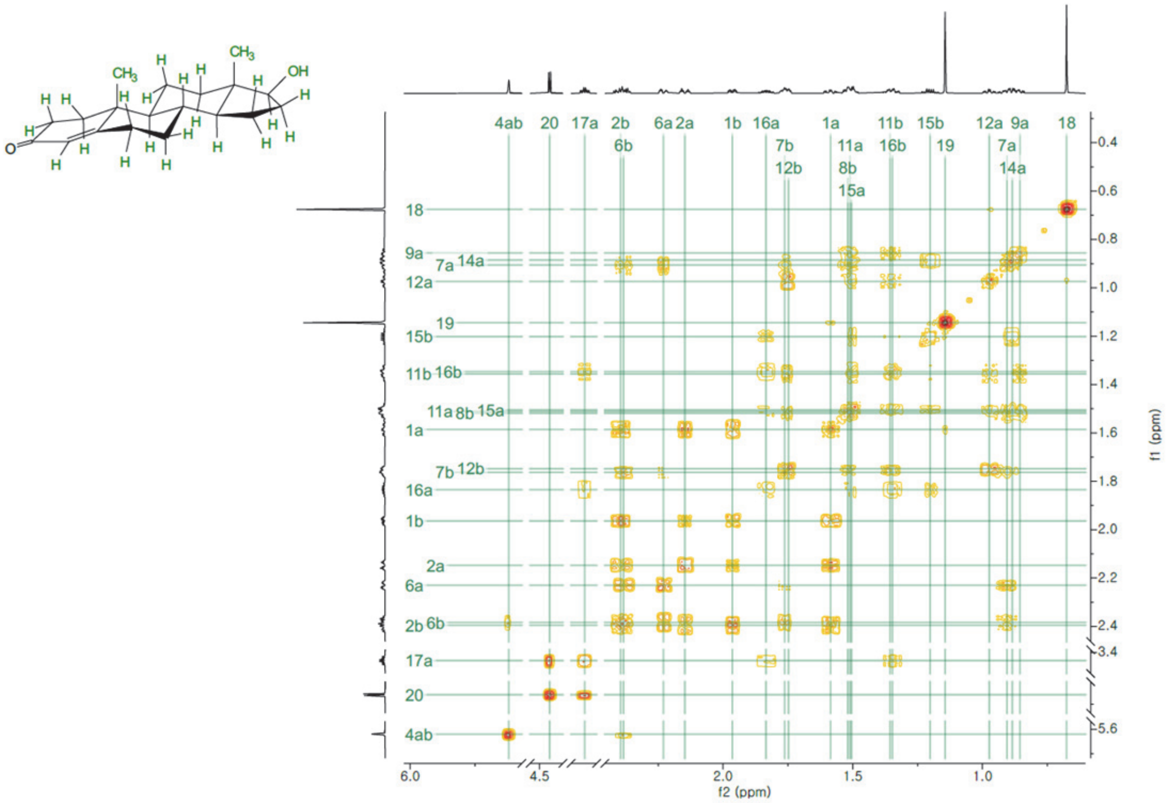

Figure S4. ^{1}H-^{1}H COSY spectra of testosterone standard.

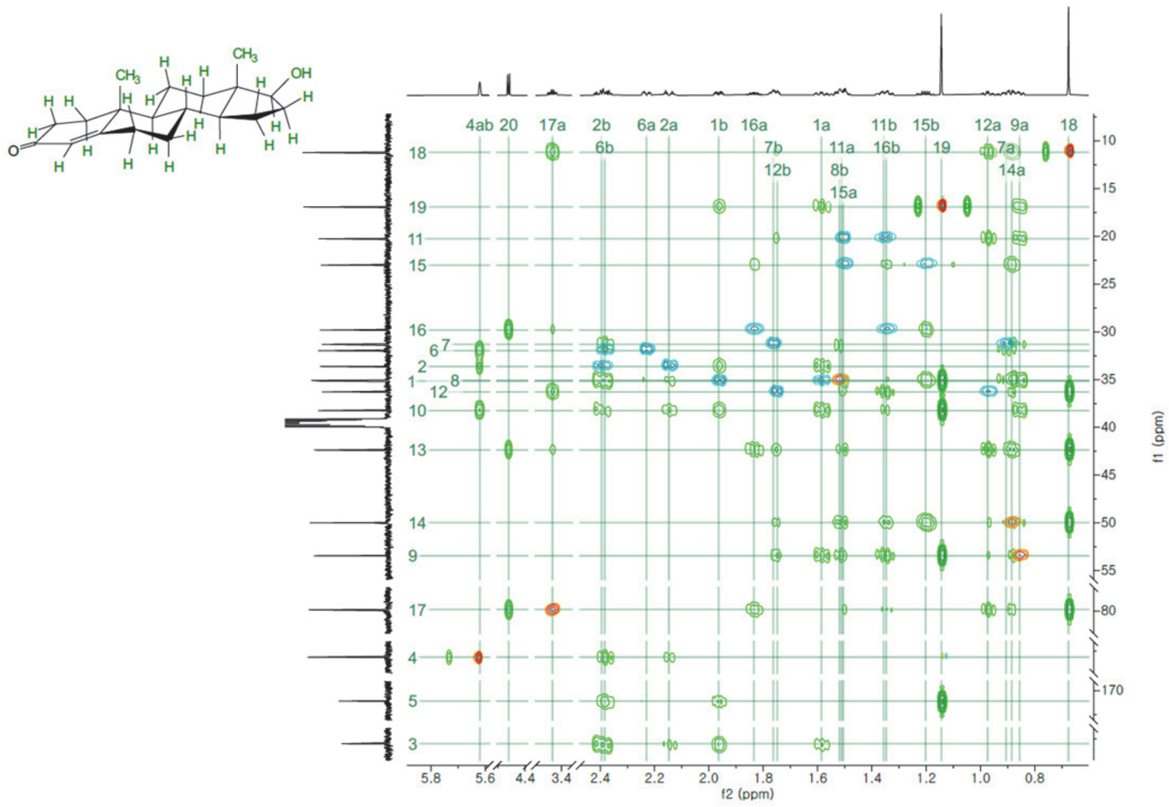

Figure S5. HSQC-DEPT and HMBC of testosterone standard were overlapped.

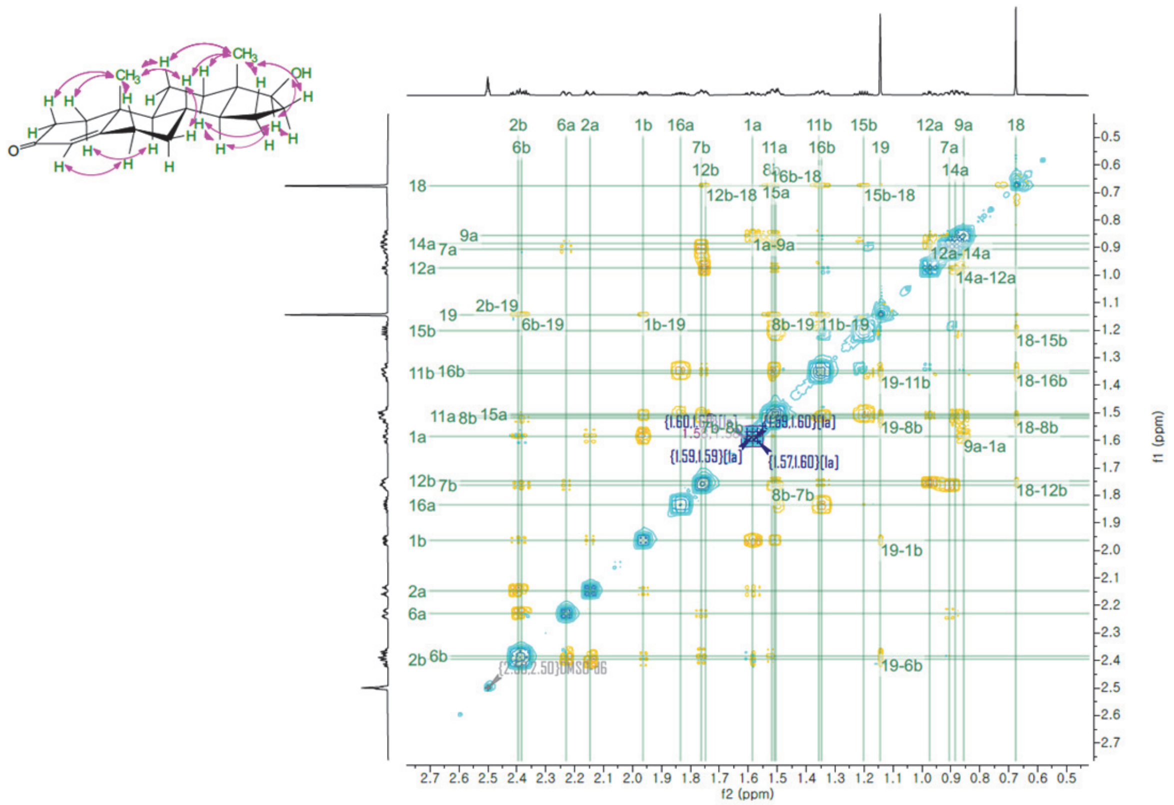

Figure S6. ^1H-^1H NOESY spectra of testosterone standard.

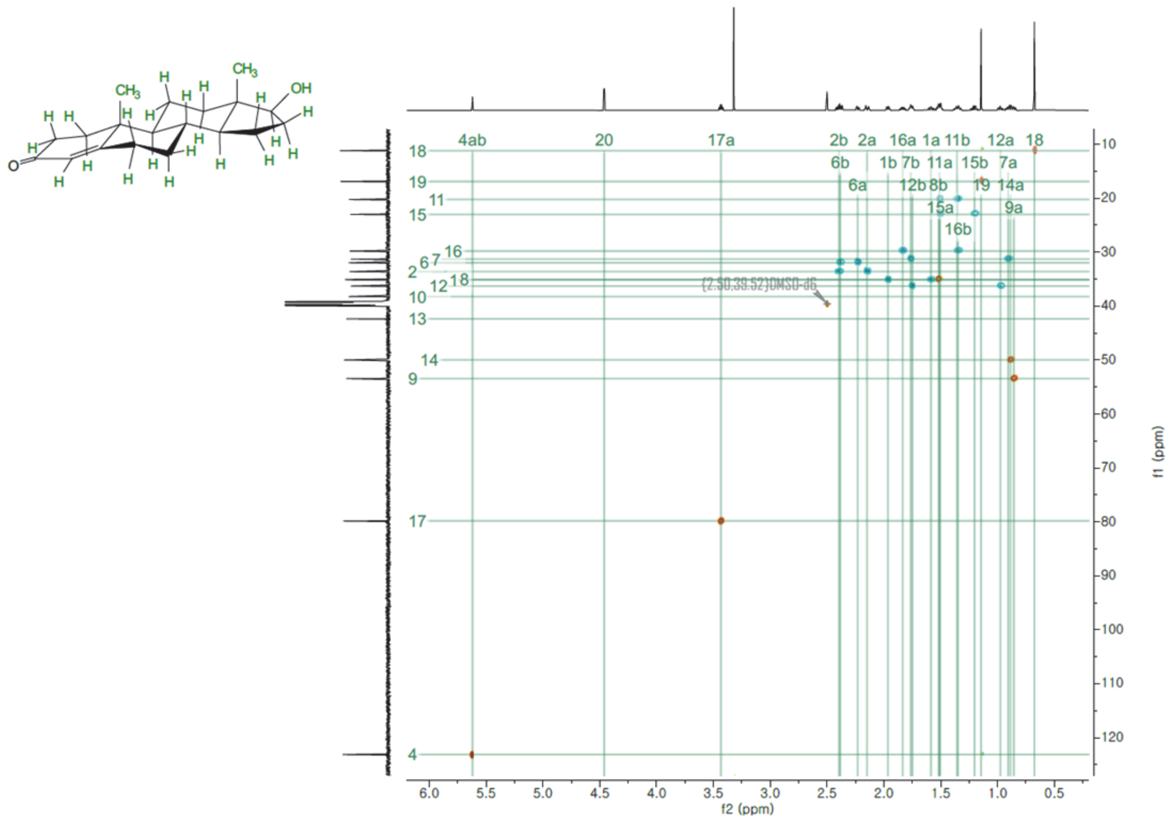

Figure S7. HSQC-DEPT spectra of testosterone standard.

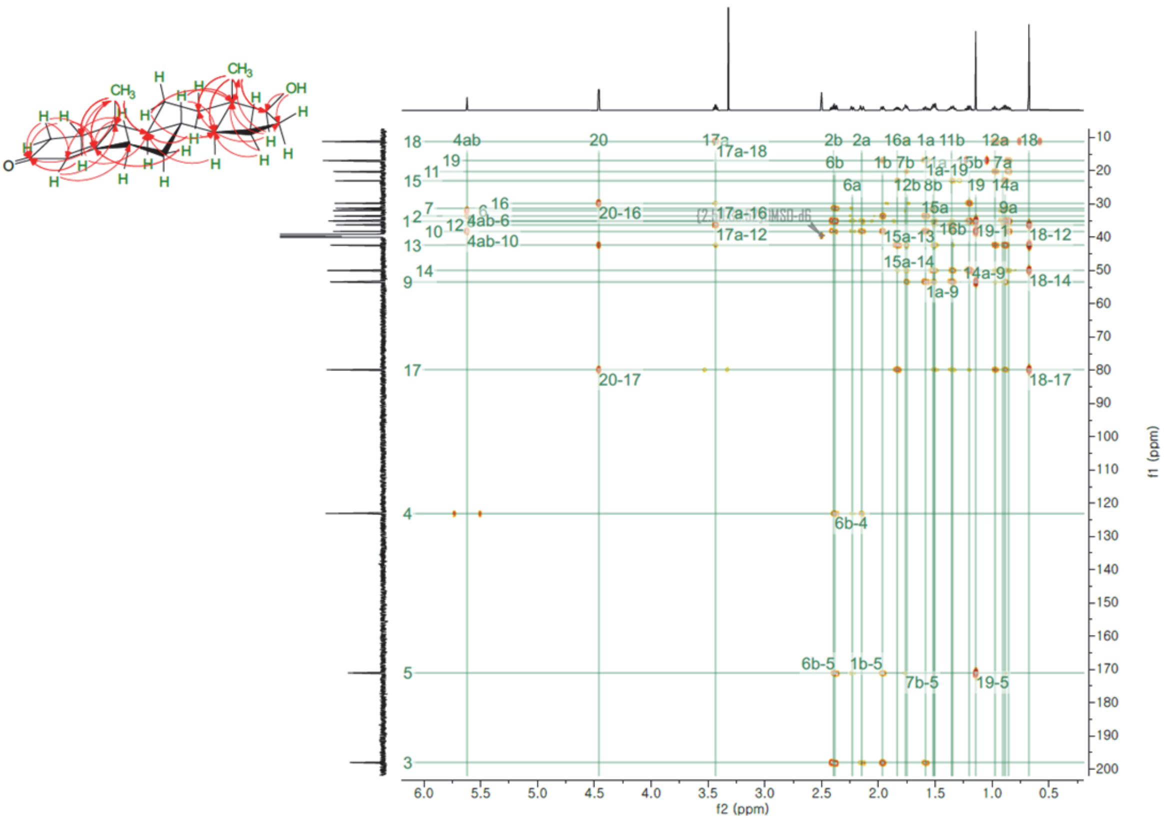

Figure S8. HMBC spectra of testosterone standard.

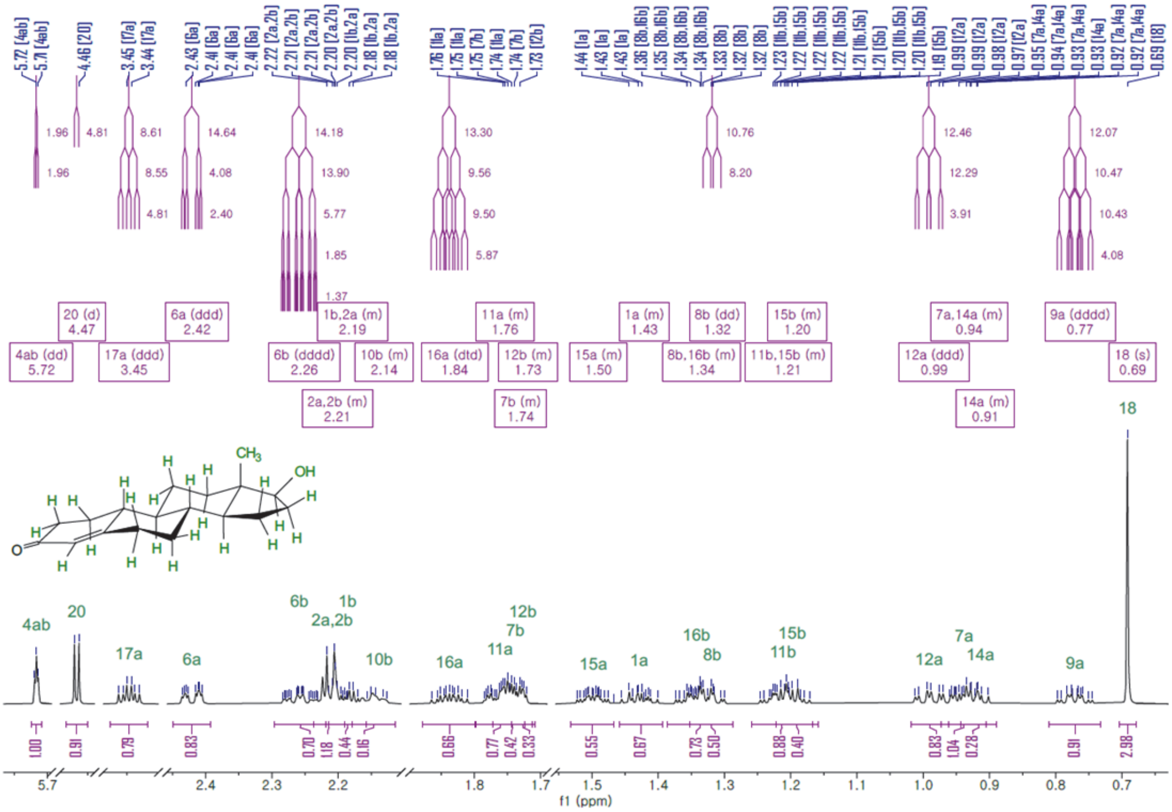

Figure S9. ^{1}H NMR spectra of nandrolone standard.

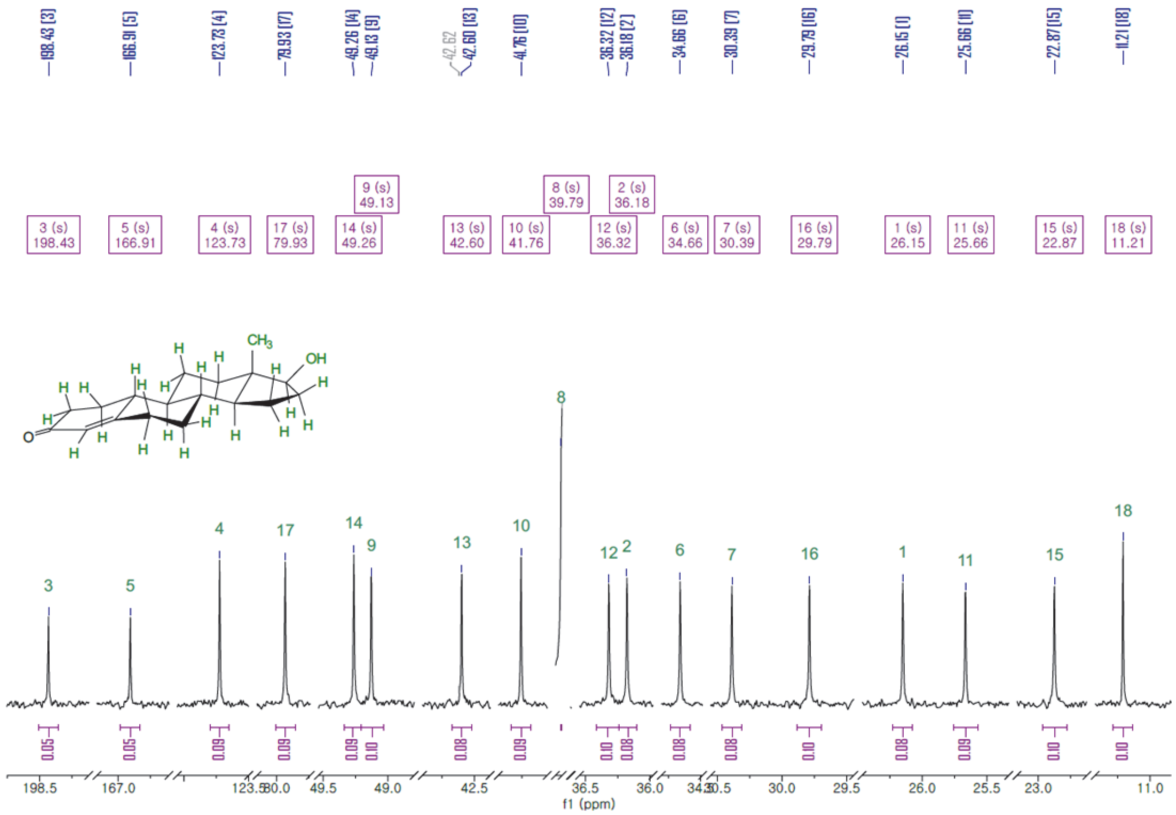

Figure S10. ^{13}C NMR spectra of nandrolone standard.

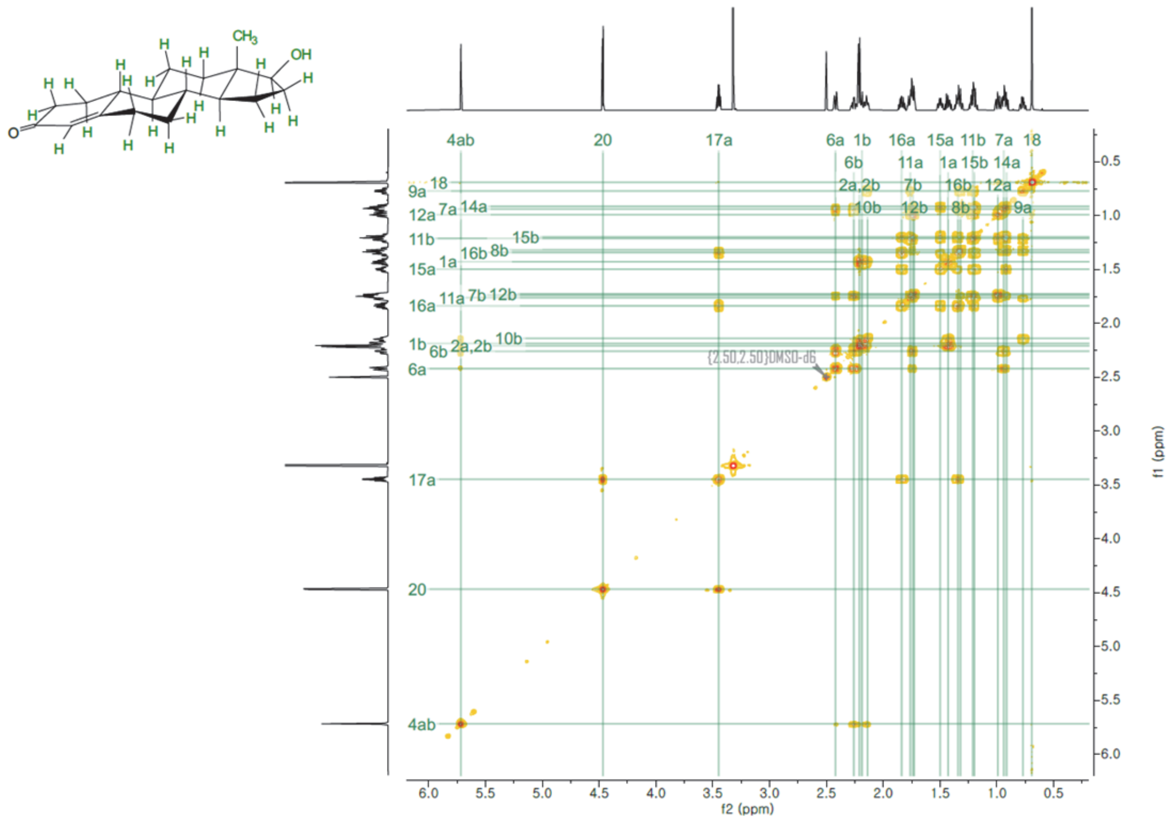

Figure S11. ^{1}H-^{1}H COSY spectra of nandrolone standard.

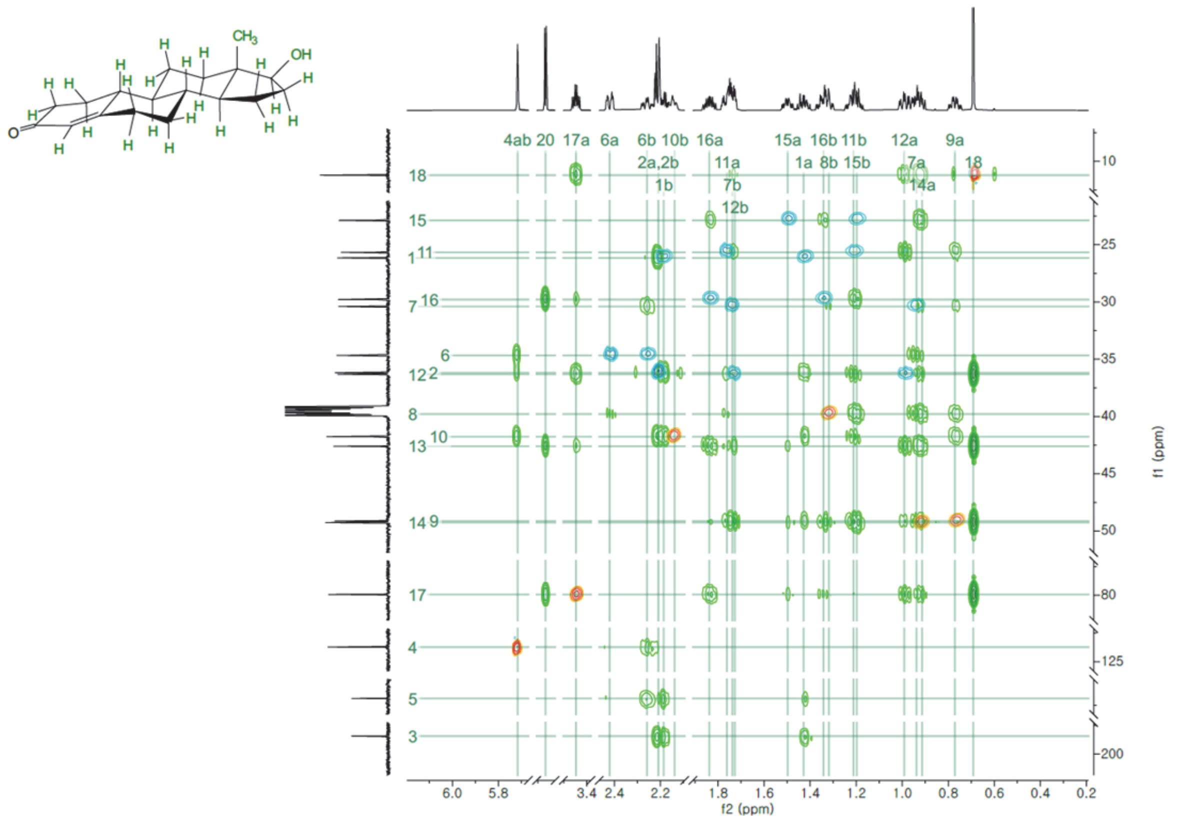

Figure S12. HSQC-DEPT and HMBC of nadrolone standard were overlapped.

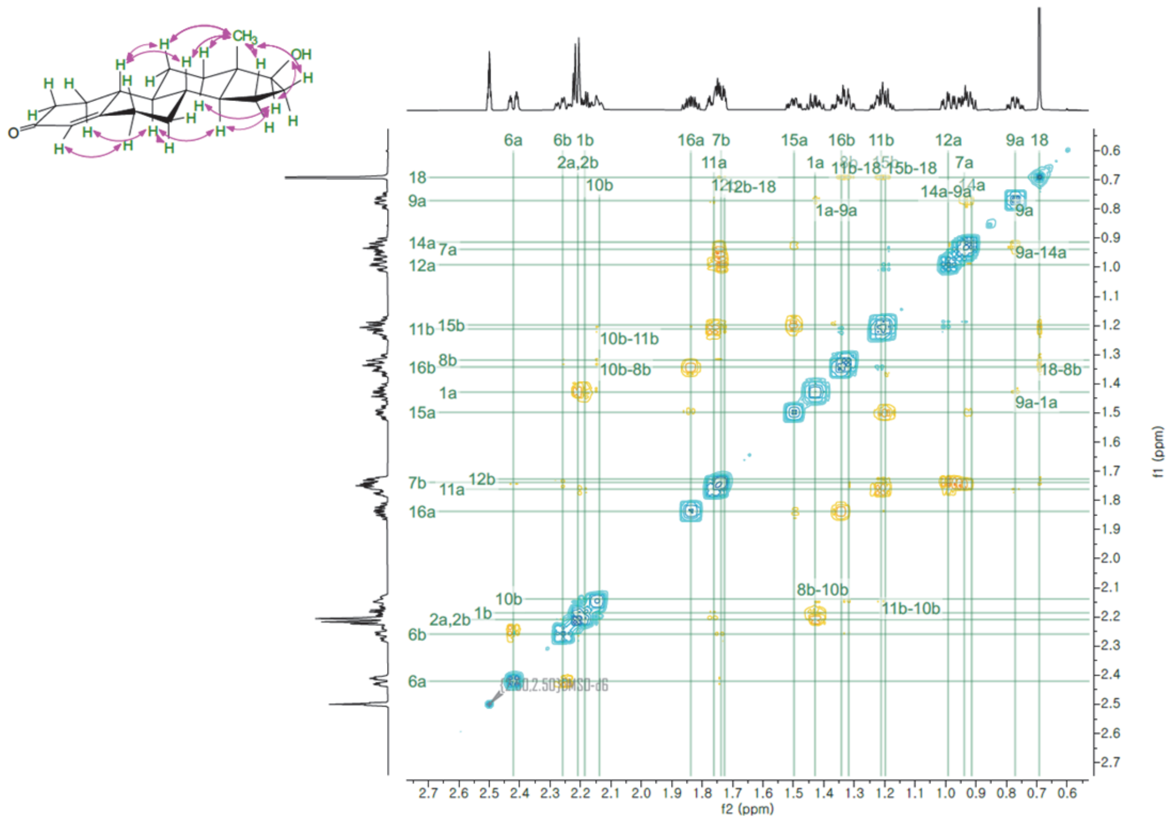

Figure S13. ^{1}H-^{1}H NOESY spectra of nandrolone standard.

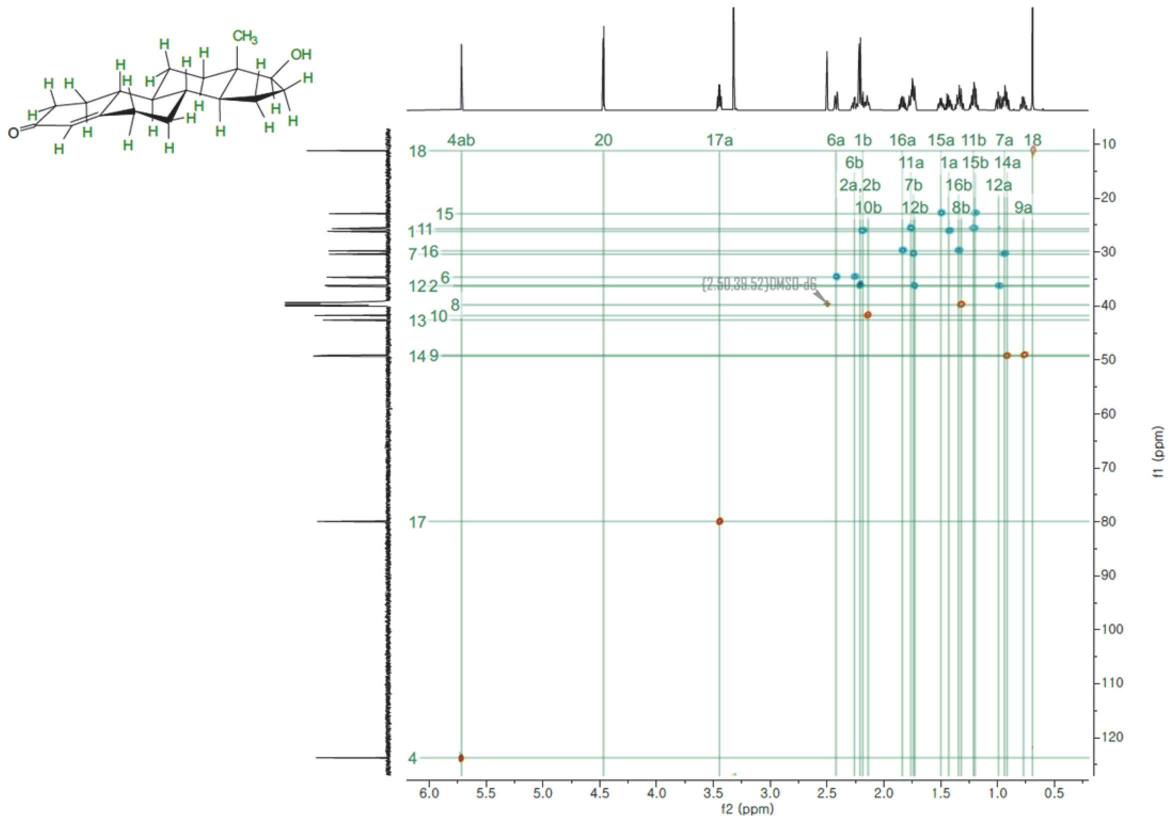

Figure S14. HSQC-DEPT spectra of nandrolone standard.

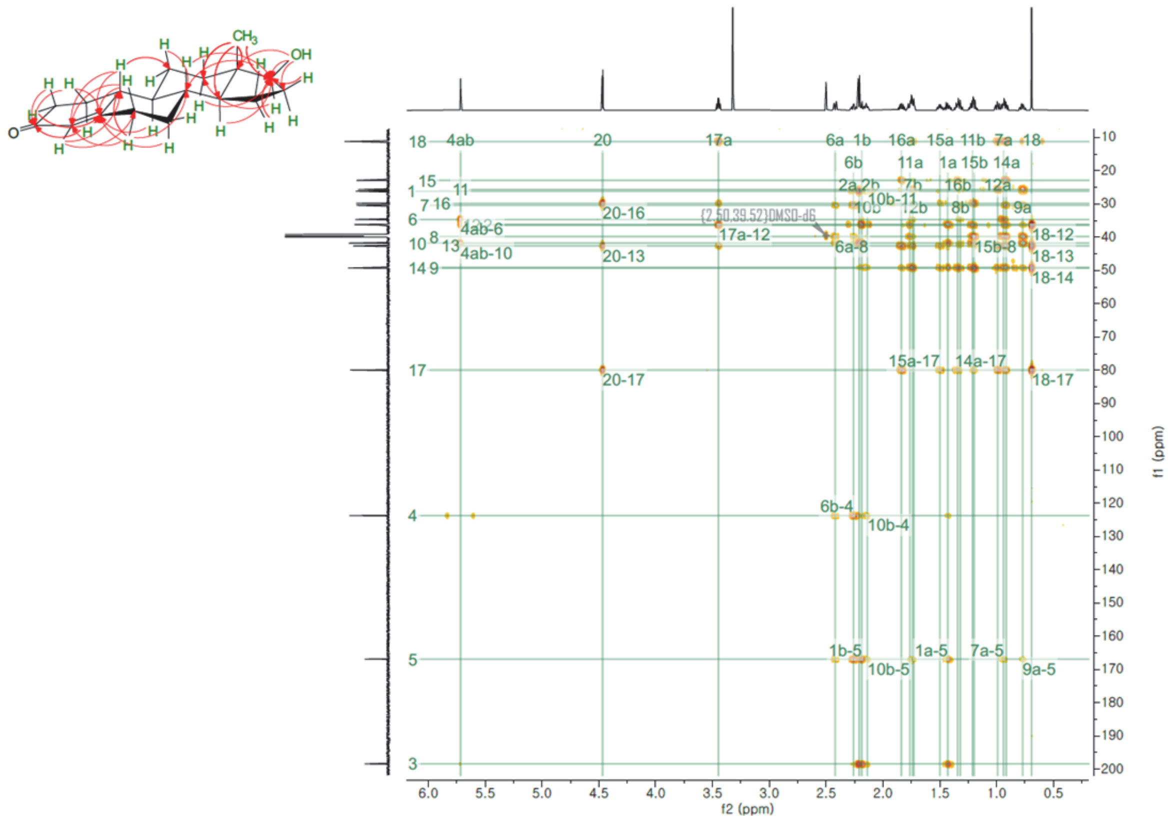

Figure S15. HMBC spectra of nandrolone standard.

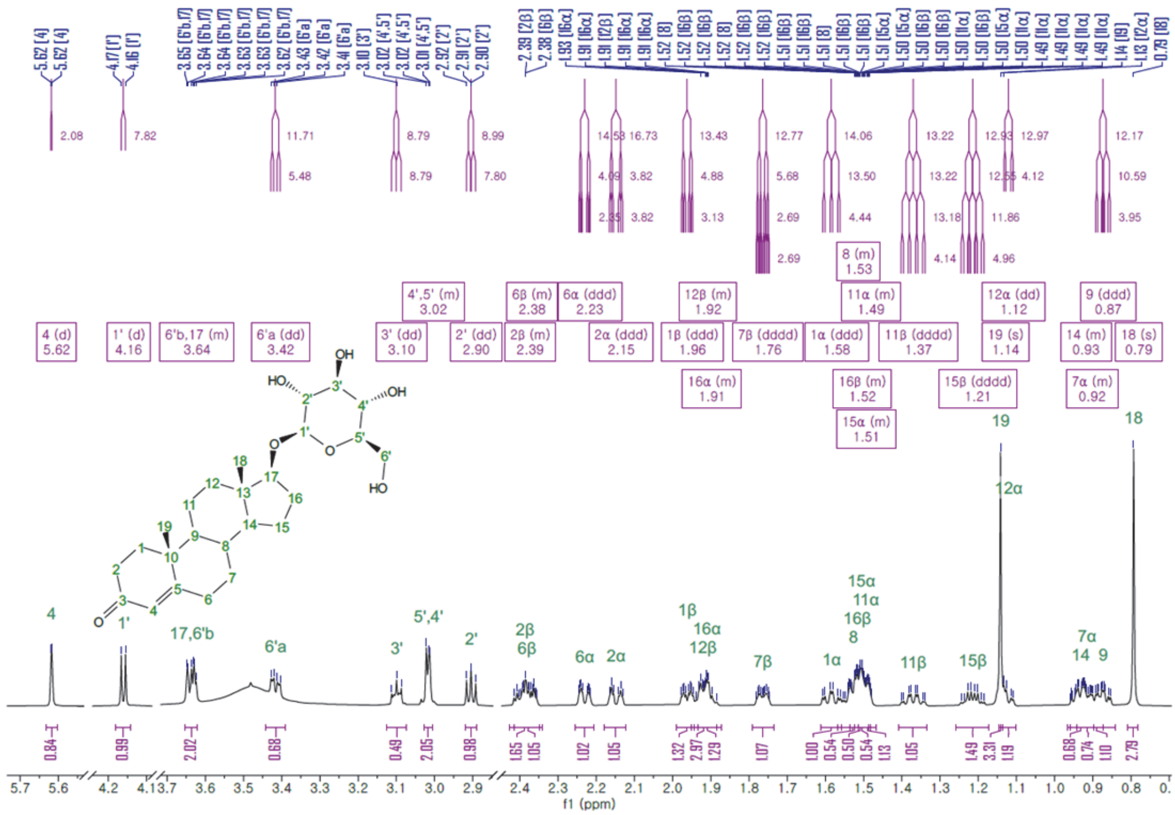

Figure S16. ^{1}H NMR spectra of testosterone mono-glucoside.

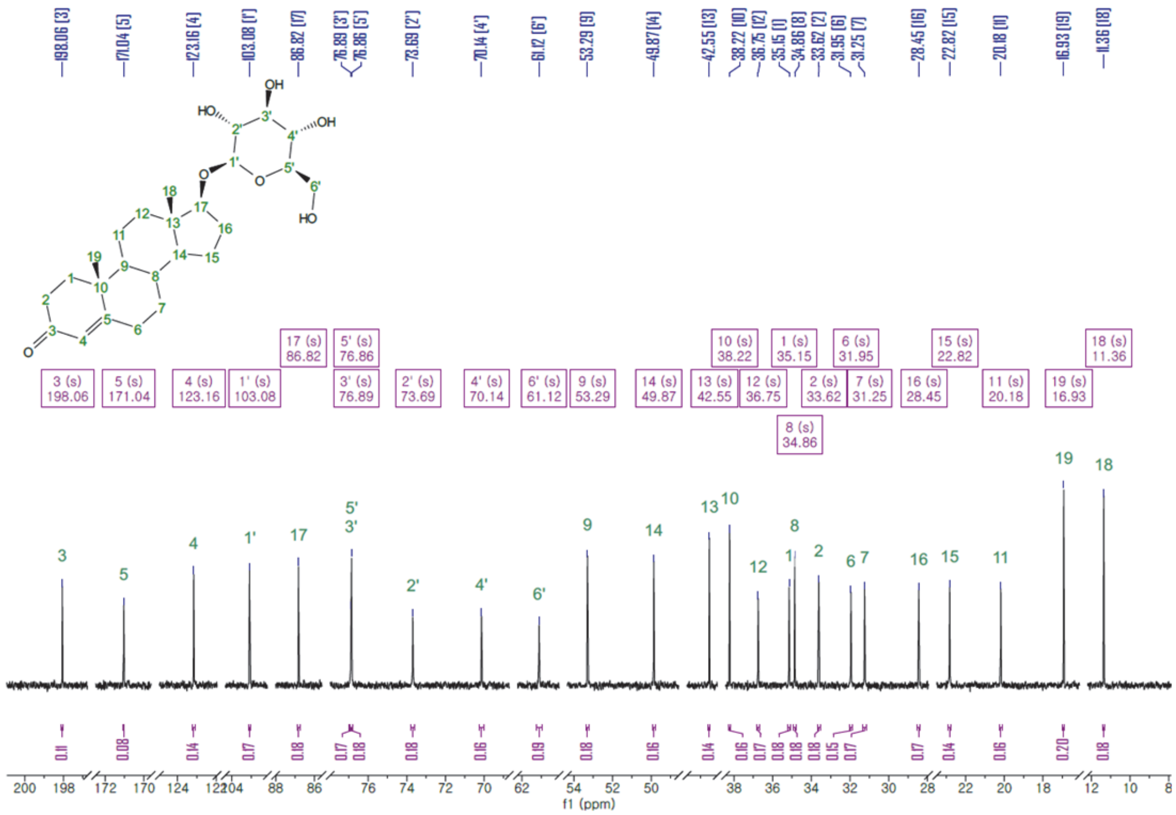

Figure S17. ^{13}C NMR spectra of testosterone mono-glucoside.

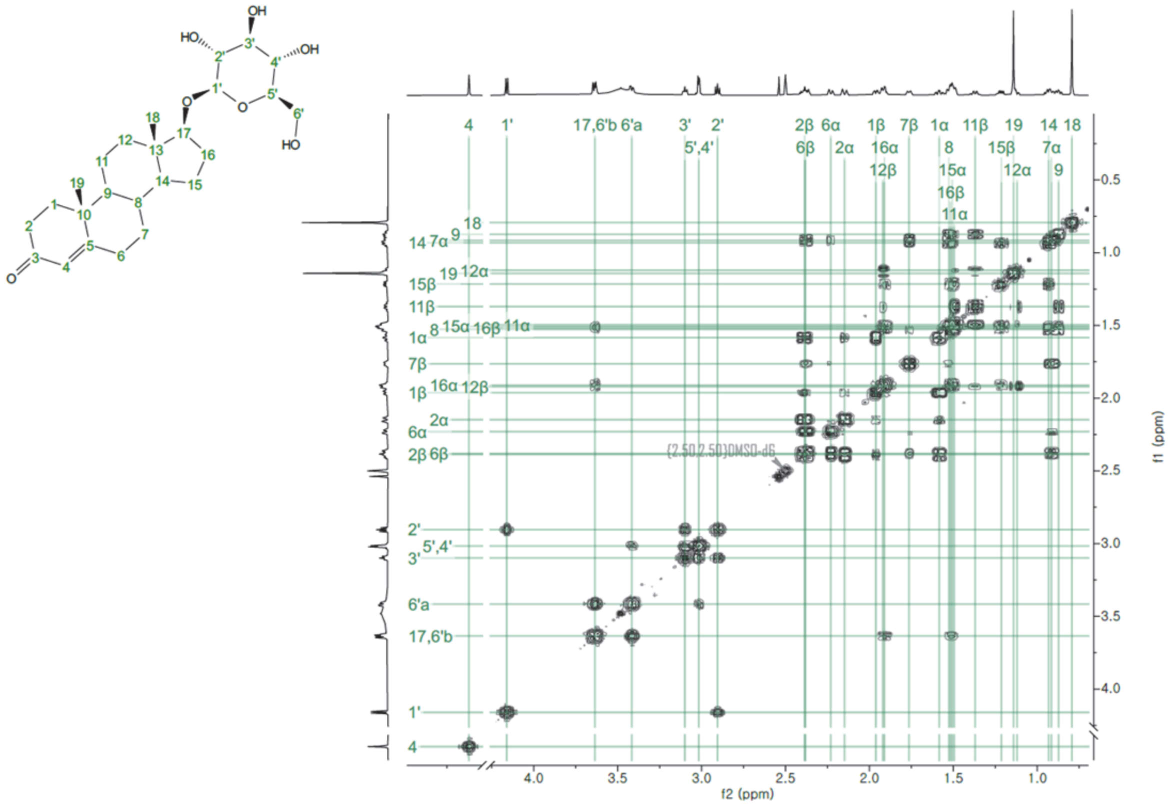

Figure S18. ^{1}H-^{1}H COSY spectra of testosterone mono-glucoside.

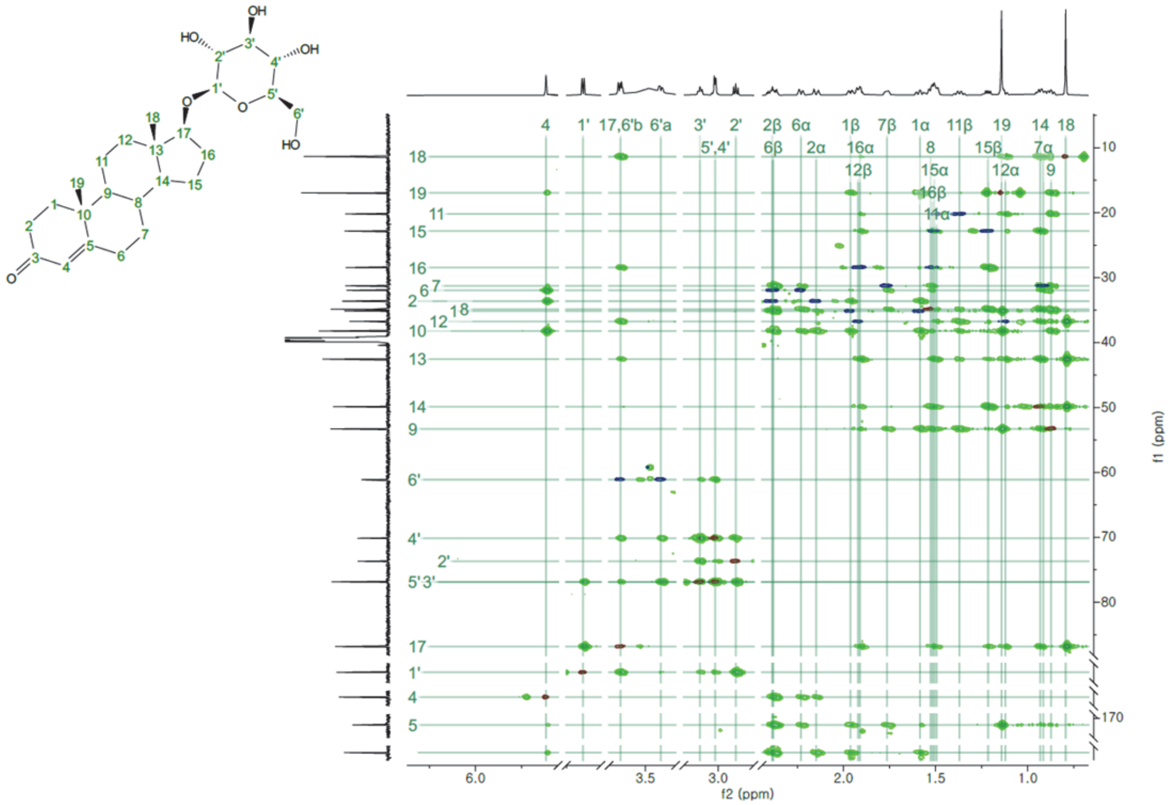

Figure S19. HSQC-DEPT and HMBC of testosterone mono-glucoside were overlapped.

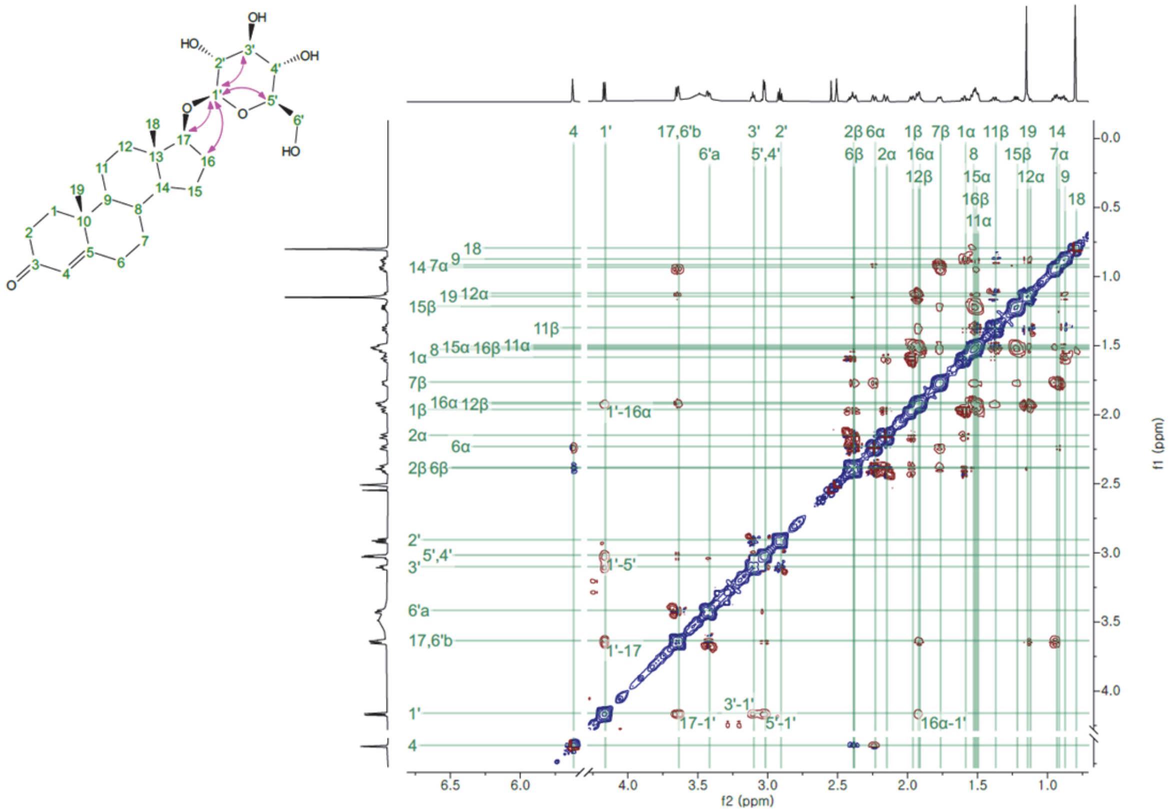

Figure S20. ^{1}H-^{1}H ROESY spectra of testosterone mono-glucoside.

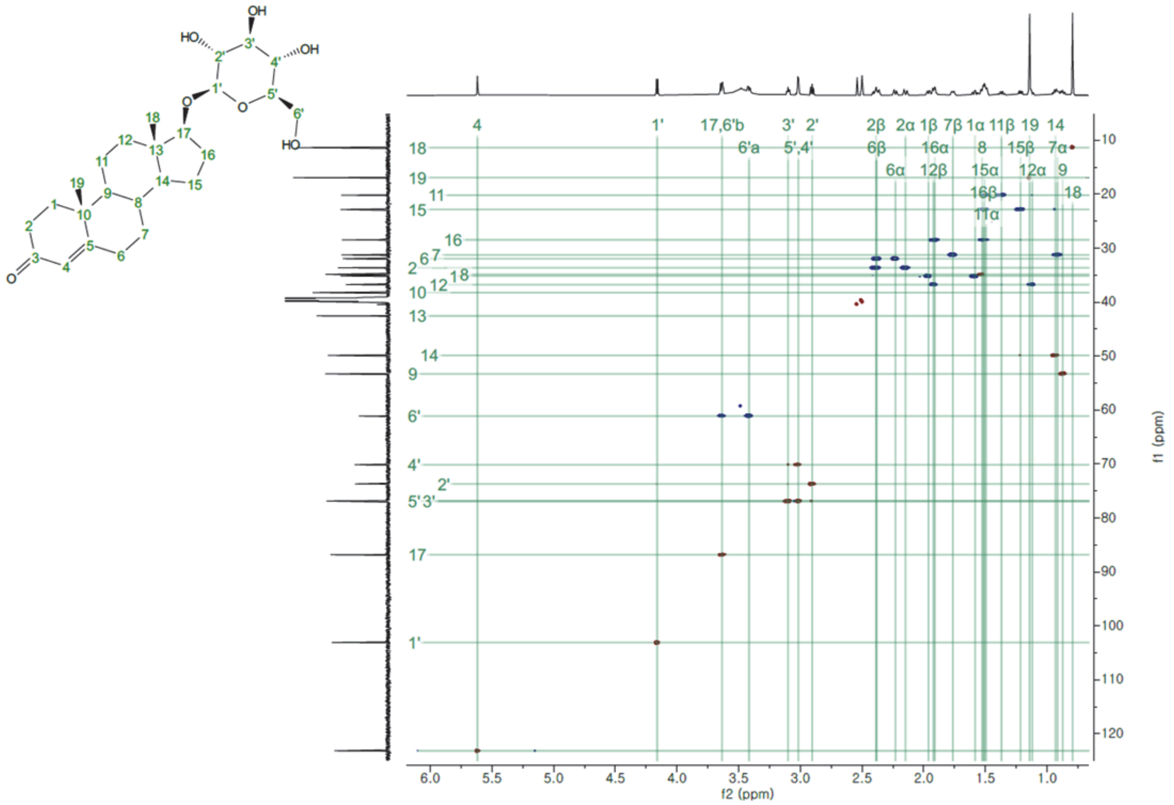

Figure S21. HSQC-DEPT spectra of testosterone mono-glucoside.

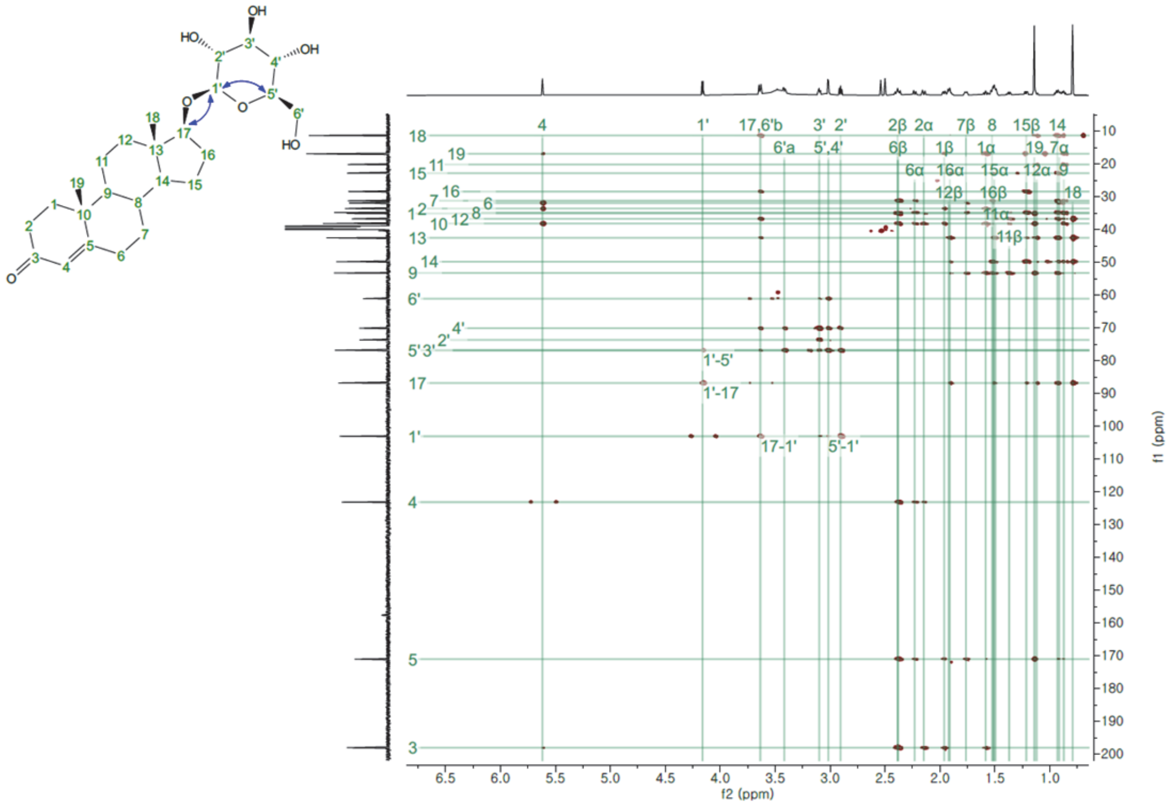

Figure S22. HMBC spectra of testosterone mono-glucoside.

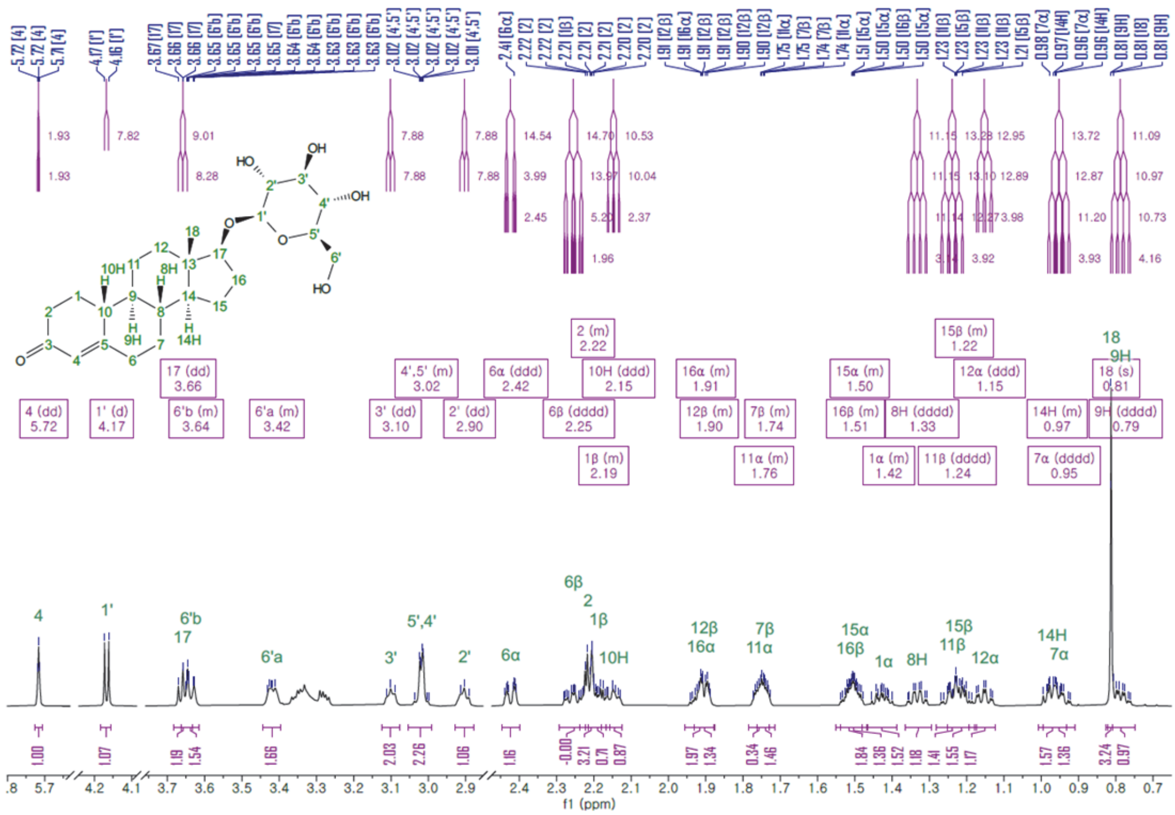

Figure S23. ^{1}H NMR spectra of nandrolone mono-glucoside.

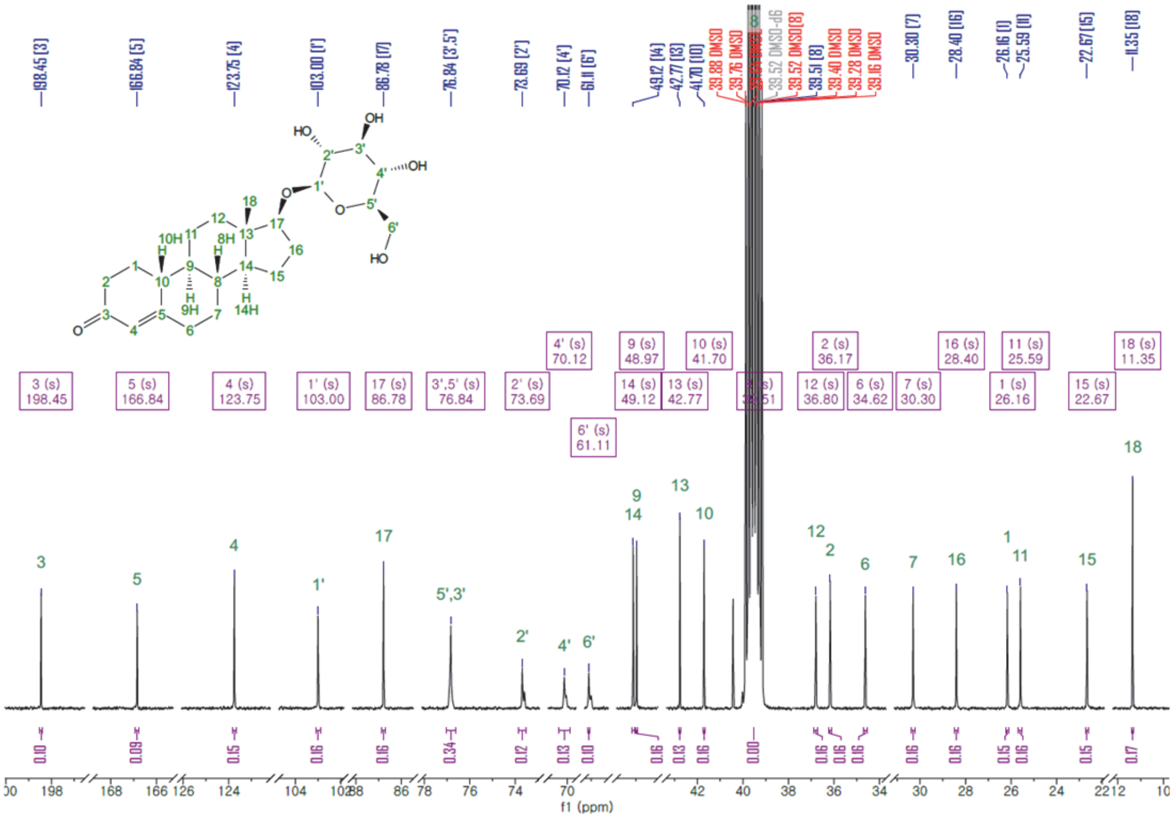

Figure S24. ^{13}C NMR spectra of nandrolone mono-glucoside.

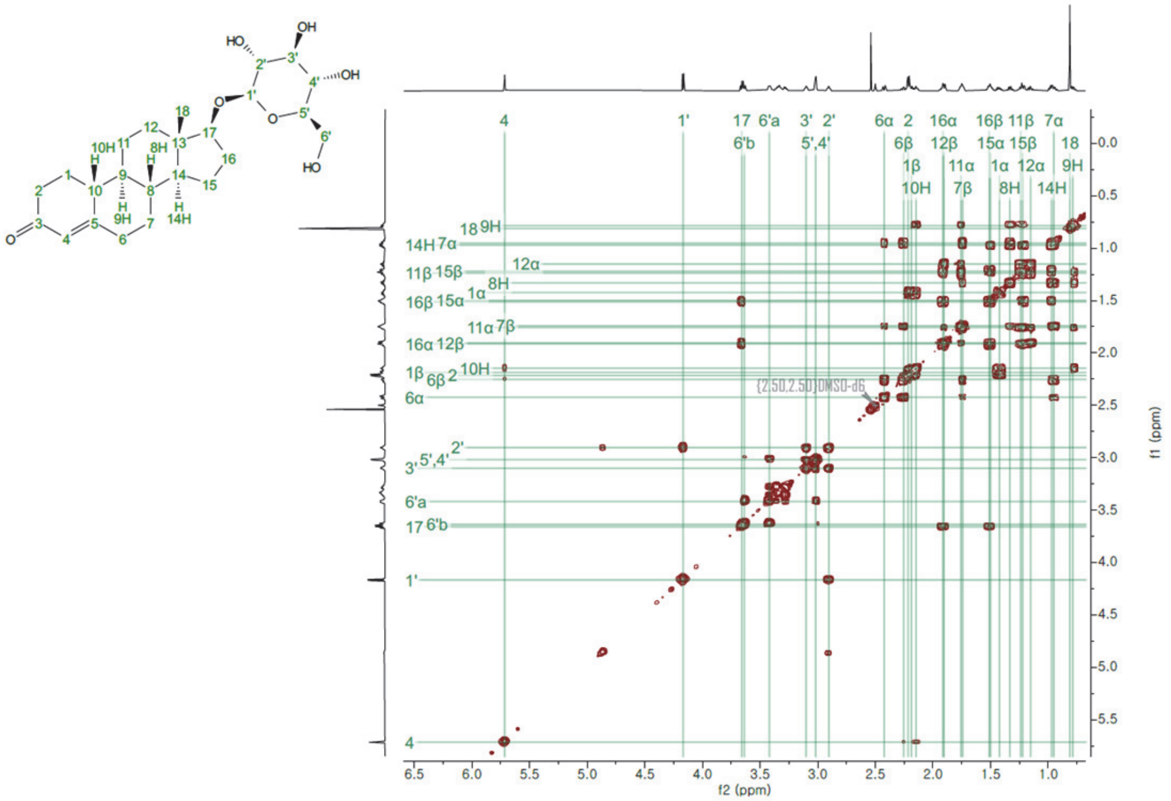

Figure S25. ^{1}H-^{1}H COSY spectra of nandrolone mono-glucoside.

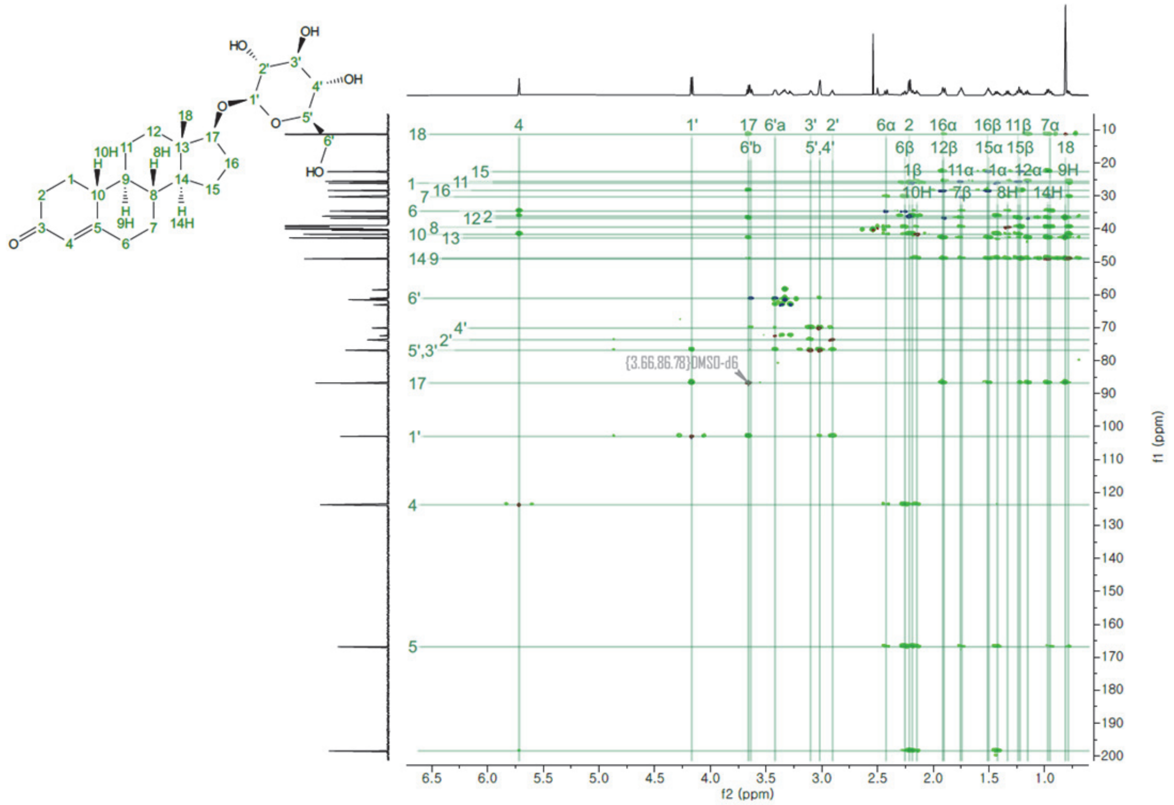

Figure S26. HSQC-DEPT and HMBC of nadrolone mono-glucoside were overlapped.

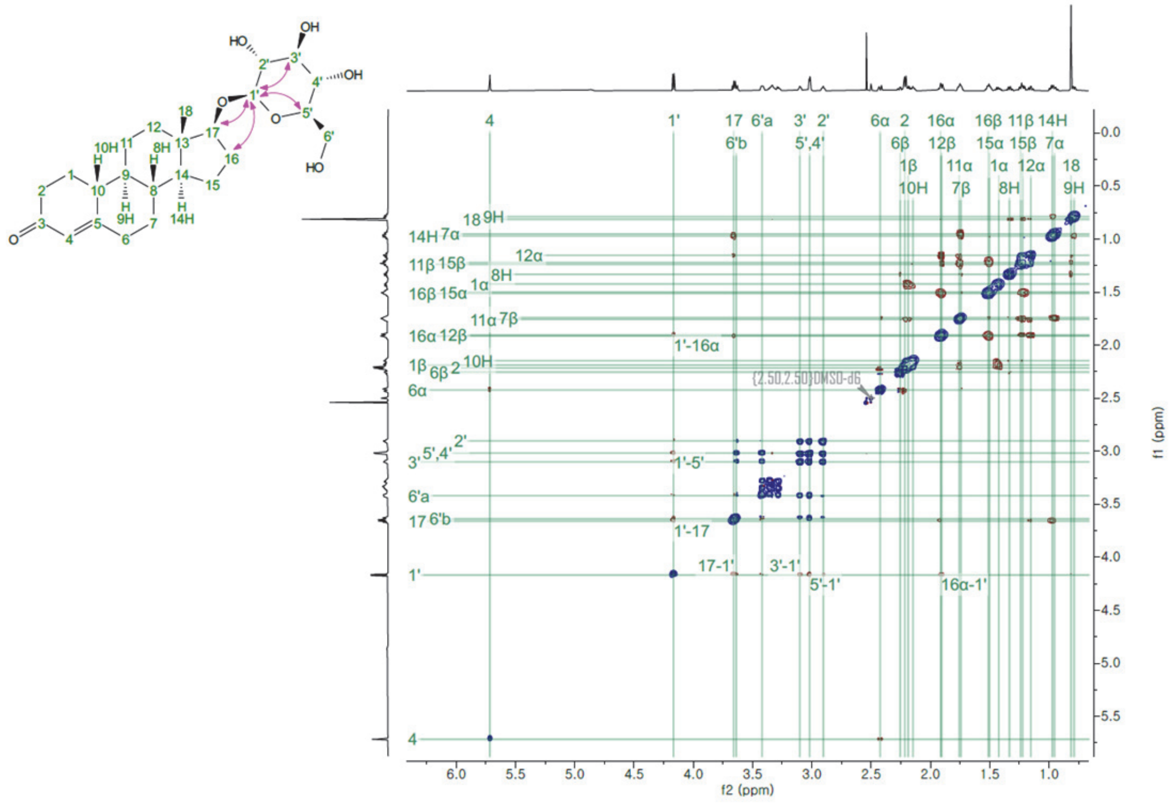

Figure S27. ^{1}H-^{1}H ROESY spectra of nandrolone mono-glucoside.

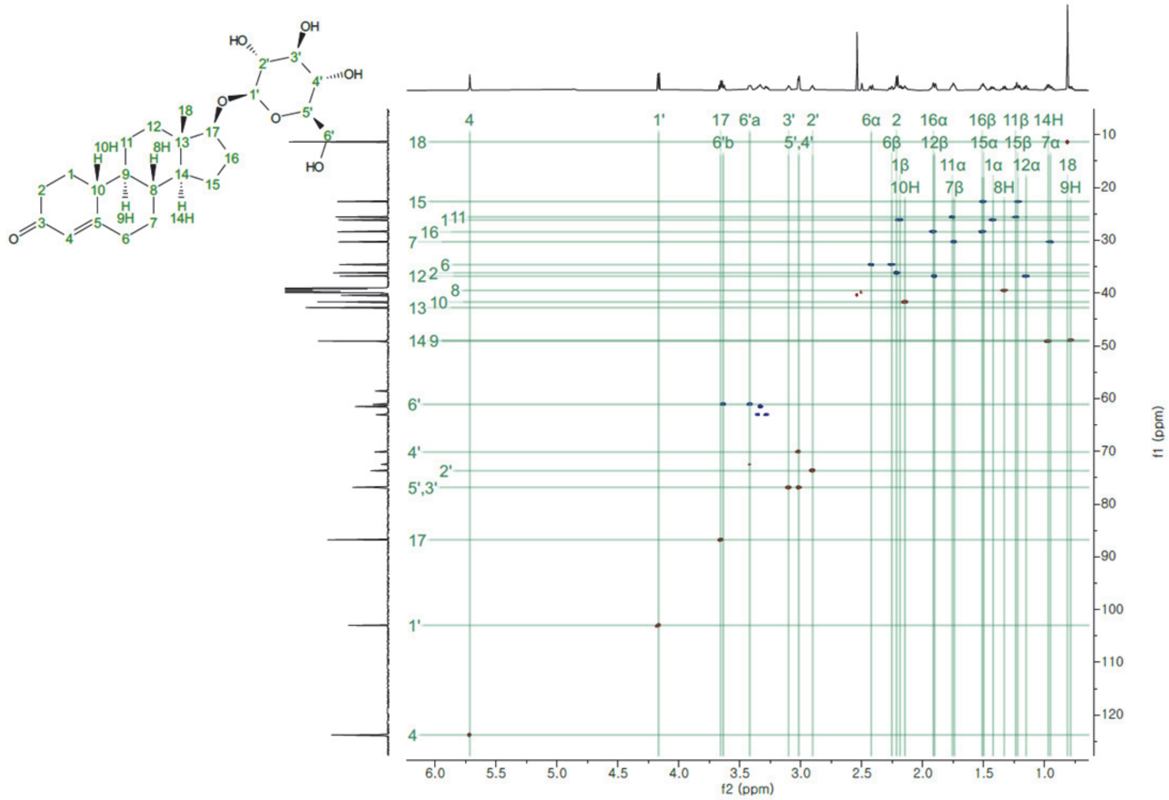

Figure S28. HSQC-DEPT spectra of nandrolone mono-glucoside.

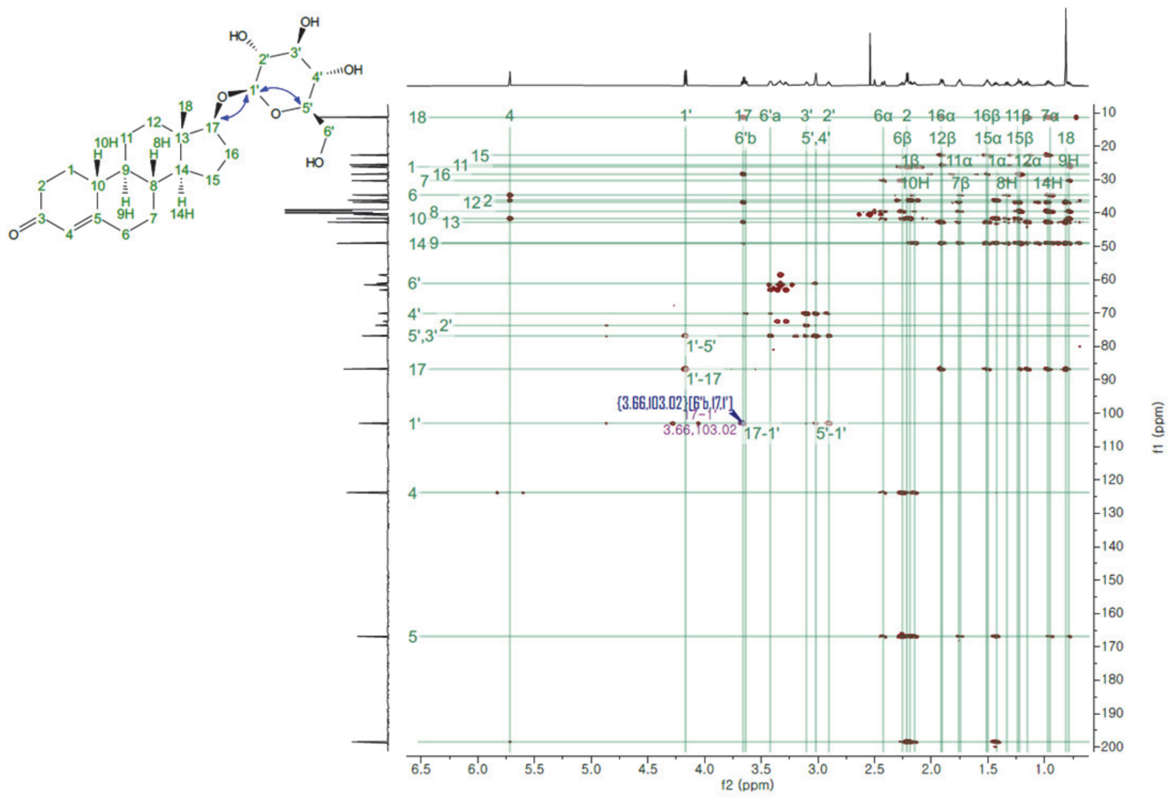

Figure S29. HMBC spectra of nandrolone mono-glucoside.
